# Genotypic detection of barriers to rat dispersal: *Rattus rattus* behind a peninsula predator-proof fence

**DOI:** 10.1007/s10530-023-03004-8

**Published:** 2023-02-06

**Authors:** Shogo Yarita, Mary Morgan-Richards, Steven A. Trewick

**Affiliations:** Wildlife and Ecology, School of Natural Sciences, Massey University, Private Bag 11-222, Palmerston North, New Zealand

**Keywords:** Aotearoa New Zealand, Microsatellite, MtDNA, Population structure, *Rattus rattus*, Wildlife sanctuary

## Abstract

Clear delimitation of management units is essential for effective management of invasive species. Analysis of population genetic structure of target species can improve identification and interpretation of natural and artificial barriers to dispersal. In Aotearoa New Zealand where the introduced ship rat (*Rattus rattus*) is a major threat to native biodiversity, effective suppression of pest numbers requires removal and limitation of reinvasion from outside the managed population. We contrasted population genetic structure in rat populations over a wide scale without known barriers, with structure over a fine scale with potential barriers to dispersal. MtDNA D-loop sequences and microsatellite genotypes resolved little genetic structure in southern North Island population samples of ship rat 100 km apart. In contrast, samples from major islands differed significantly for both mtDNA and nuclear markers. We also compared ship rats collected within a small peninsula reserve bounded by sea, suburbs and, more recently, a predator fence with rats in the surrounding forest. Here, mtDNA did not differ but genotypes from 14 nuclear loci were sufficient to distinguish the fenced population. This suggests that natural (sea) and artificial barriers (town, fence) are effectively limiting gene flow among ship rat populations over the short distance (~ 500 m) between the peninsula reserve and surrounding forest. The effectiveness of the fence alone is not clear given it is a recent feature and no historical samples exist; resampling population genetic diversity over time will improve understanding. Nonetheless, the current genetic isolation of the fenced rat population suggests that rat eradication is a sensible management option given that reinvasion appears to be limited and could probably be managed with a biosecurity programme.

**Supplementary Information:**

The online version contains supplementary material available at 10.1007/s10530-023-03004-8.

## Introduction

Three species of rat (*Rattus rattus*, *R. norvegicus, R. exulans)* are troublesome pests in Aotearoa New Zealand (Tompkins 2018), and problematic around the globe, transmitting human diseases and damaging food resources (Gilabert et al. 2007; Sonne 2016; Strand and Lundkvist 2019). The three rat species in New Zealand were introduced by humans and are recognized as environmental pests due to their negative effect on native fauna and flora—eating birds, seeds, snails, lizards, fruit, insects, eggs, larvae and flowers (Daniel 1973; Innes 1979; Wilmshurst and Carpenter 2020; Wolf et al. 2018). Norway rat (*R. norvegicus*) and ship rat (*R. rattus*) were introduced from Europe and Asia in the eighteenth and nineteenth centuries (Atkinson 1973; King 2019), and although both are widespread in New Zealand, the ship rat is the most common species. This contrasts with the situation in Europe where Norway rats are considered the major problem species because of their prevalence in urban environments. An ambitious plan to eradicate seven invasive predatory mammal species from New Zealand by 2050 (Murphy et al. 2019) includes ship rat, which has been the target of population control over of many years with several successful eradications from offshore islands and fenced reserves in the New Zealand archipelago (Brown et al. 2015; Russell and Broome 2016). The reappearance of rats in reserves after eradication (Fewster et al. 2011; Russell et al. 2009, 2010) and the challenges of scaling management across 268,000 km^2^ indicates that a step-change in approach will be required if the 2050 target is to be achieved. Specifically, eradication efforts would benefit from knowing how effective natural and artificial landscape features are as barriers to rat dispersal.

The re-emergence of pest populations results either from undetected individuals that survive eradication efforts, or reinvasion from an adjacent habitat (Russell et al. 2008; McMillan and Fewster 2017; Richardson et al. 2019). Even low density populations can be subject to detectable dispersal by juvenile and adult rats over land (Hansen et al. 2020) and the swimming ability of rats means there is even a risk of reinvasion for some offshore reserve islands (Russell et al. 2008). The success with which rats stowaway on boats and other human transport (Wilmshurst et al. 2008) means that any barrier crossed by humans might also be permeable to rats. Nevertheless, a combination of natural and artificial dispersal barriers might reduce the cost of establishing reserves, and peninsulas are obvious candidates for this approach (Burns et al. 2012; Innes et al. 2019).

In the absence of direct observations of individual rat activity, the origins and composition of pest populations are best identified using population genetic approaches (Beaumont 1999; Desvars-Larrive et al. 2019). As recent experience with SARS-CoV-2 among the human population has shown (e.g. Park et al. 2021), understanding the pathways of pest invasion requires genotypic information that can provide identification of populations and even individual lineages (Blair 1953; Browett et al. 2020). Spatial separation limits interbreeding and results in different proportions of genetic variants (alleles) in isolated populations, and the disparity in allele frequencies can be enhanced by genetic drift in small populations (Wright 1931; Frankham 1996). Comparison of allele frequencies can therefore distinguish among populations and reveal the pattern and extent of dispersal (gene flow) (e.g. in rodents Fewster et al. 2011; Bradley et al. 2017; Russell et al. 2019; Richardson et al. 2019).

Population genetic tools allow scrutiny of pest population dynamics and so can inform management strategy by revealing metapopulations and their demography, migration rates and routes (Rollins et al. 2006) even at small spatial scales (Combs et al. 2019). Here we applied PCR based genotyping to densely sampled ship rats in central New Zealand to assess the sensitivity of these markers for monitoring and modelling rat populations. We examine Cook Strait as a barrier to rat dispersal between North Island and South Island and we assess the capacity of a predator-proof fence and natural water barrier on a small peninsula in a suburban location to limit ship rat gene flow. If the rat population within a wildlife sanctuary of this type is genetically differentiated from the surrounding rat population, it would suggest that population suppression within the reserve can be maintained and eradication feasible.

## Materials and methods

### Location

**Fig. 1:**
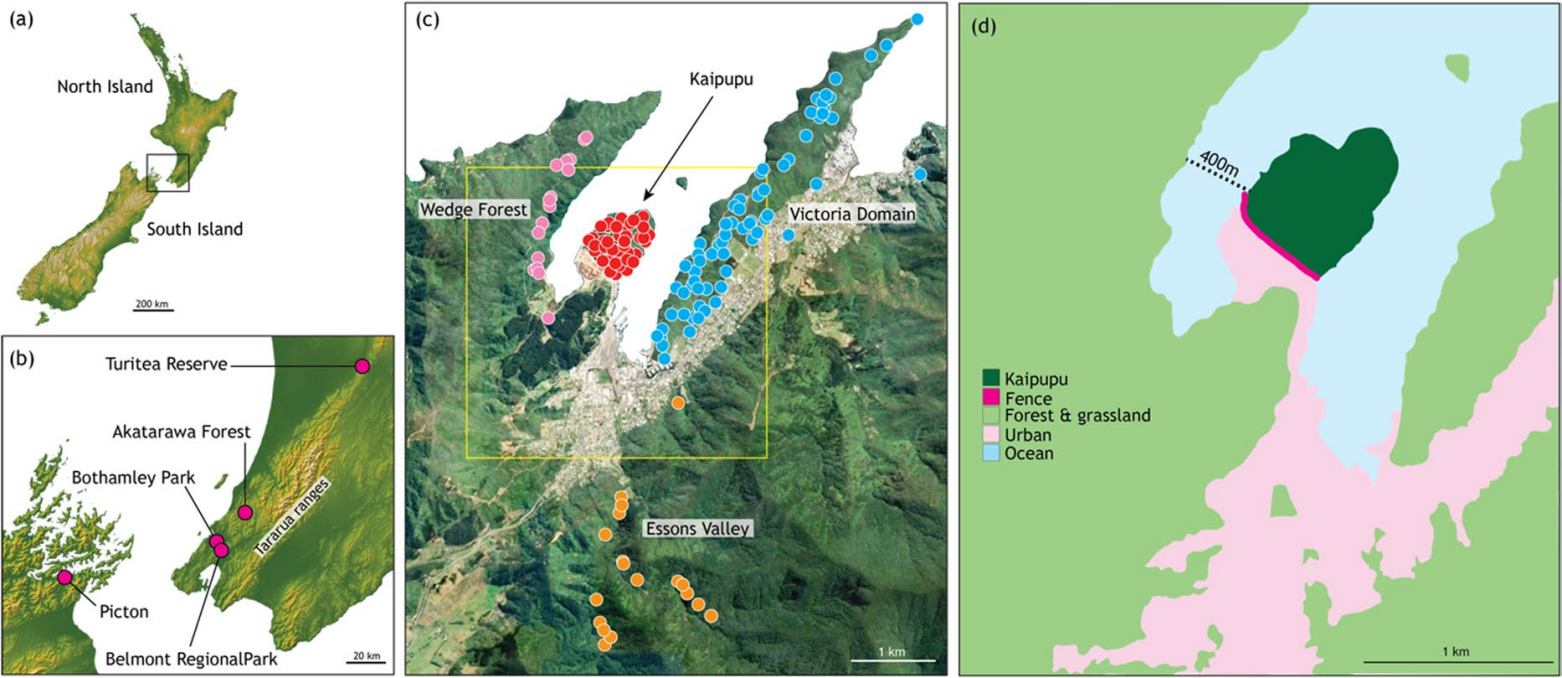
Sampling locations for ship rat (*Rattus rattus*) in central New Zealand (**a**). Southern North Island and northern South Island (**b**). Fine scale sampling locations at Picton, South Island where each point is a trap location (**c**). Environment in the vicinity of Kaipupu (yellow square in c) (**d**)

Kaipupu Wildlife Sanctuary (Kaipupu) is at the entrance to Picton Harbour, adjacent to the port where timber for export is stored. It is on a small peninsular but the adjacent shore is < 500 m away (Fig. 1), which is within the plausible swimming range of ship rats (Russell et al. 2008). The native forest fragment, known as Kaipupu was established as a sanctuary in 2005 and a 600 m predator-proof fence at its land boundary that extends below low-water was completed in 2010. The fence was directed at preventing incursions of rats and stoats, and within Kaipupu pest trapping has been undertaken since inception of the reserve to suppress and monitor population status. In consideration of native wildlife the community opted to exclude use of toxins within the reserve and with a sufficiently high trap density it was predicted that pest numbers would dwindle within the reserve if the fence was an effective barrier. At the same time trapping was maintained in surrounding forest and suburban habitat (Wedge Forest, Victoria Domain, Essons Valley; Fig. 1). Kaipupu trapping effort increased with more than 500 ship rats caught in 2019. The present analysis used tails from rodents trapped by the Picton Dawn Chorus between 2017 and 2020, all within 8.6 km of one another. Sampling locations were mapped using QGIS 3.16.13 (QGIS Development Team 2021).

Most shipping between North Island and South Island comes through the port in Picton Harbour. Therefore if rats were moving between islands, we would expect to detect gene flow from North Island rats among the Picton population samples. Across Cook Strait, in southern North Island, rat populations were sampled from four forested locations within 105 km of one another during 2016 and 2017 (Sran 2019): Akatarawa Forest, Bothamley Park, Turitea Reserve and Belmont Regional Park (Fig. 1). Tissue samples consisted of approximately 10 mm of tail tip stored in 95% ethanol with a record of trap location (GIS) and date (Supplementary Table S1).

### Genetic markers

DNA was extracted from rodent tails using the Extracta™ DNA Prep for PCR—Tissue (Quanta BIOSCIENCES) following the manufacturers protocol. A tenfold dilution of the resulting DNA solution was used for subsequent polymerase chain reaction (PCR) amplification. Amplification of partial D-loop (585 bp) from the mitochondrial genome used the primers: EGL4L 5′-CCACCATCAACACCCAAAG-3′ and RJ3R 5′- CATGCCTTGACGGCTATGTTG-3′ (Robins et al. 2007). Amplification reactions were in 10 μL volumes with final concentrations of 1 × DreamTaq Green buffer (ThermoFisher Scientific), 200 μM each deoxy-nucleotide phosphates, 0.25 μM each primer (EGL4L and RJ3R), and 0.05U DreamTaq DNA polymerase (Thermo Fisher Scientific). Thermocycling used an initial denaturation step of 94 °C for 5 min; 35 cycles of 94 °C for 30 s, 60 °C for 30 s, 72 °C for 1 min with a final extension step of 72 °C for 10 min. Amplicons were sequenced using Big-Dye^®^ chemistry (Perkin Elmer) following the manufacturer’s protocol (ABI3730 DNA analyser; Macrogen Inc).

The same DNA samples were used for genotyping. Initially, 20 microsatellite loci were selected for genotyping the rats. Primers for ten of these loci were originally developed for Norway rat (D2Rat234, D10Rat20, D16Rat81, D7Rat13, D20Rat46, D19Mit2, D5Rat83, D15Rat77, D11Mgh5 and D18Rat96; Jacob et al. 1995), but have been successfully used to study ship rat (Abdelkrim et al. 2010; Gatto-Almeida et al. 2020; Miller et al. 2010; Russell et al. 2010). Another ten microsatellite loci specifically developed for ship rat (Rr14, Rr17, Rr21. Rr22, Rr54, Rr67, Rr68, Rr93, Rr107 and Rr114; Loiseau et al. 2008) were also screened.

Multiplex PCR was carried out using the *QIAGEN* Multiplex PCR Kit with three multiplexes of primers labelled with a combination of three fluorophores (Table 1). Reaction volumes of 10 µL included 5 µL of 2 × QIAGEN Multiplex PCR Master Mix, 1 µL of 100 µM Primer Mix (containing each primer at 2 µM), 3 µL of distilled water and 1 µL of 10 × diluted DNA extraction. The PCR regime was an initial denaturation step of 95 °C for 15 min; 30 cycles of 94 °C for 30 s, 55 °C for 90 s, 72 °C for 1 min with a final extension step of 60 °C for 30 min. Amplicons were genotyped using a fragment analyser at Macrogen Inc, with GeneScan 500 LIZ size standard (Applied Biosystems).

**Table 1:** Three microsatellite primer multiplex combinations (Mplex) used for ship rat (*Rattus rattus*) genotyping

Locus	Primer sequence (5′-3′)	Source	Fluorophore	Size range (bp)	Mplex
Rr68^#^	F: GACTTCCTATCCAGACAGAG R: CTGAAGCTATAAAGTGAGATCTA	Loiseau et al. (2008)	FAM	105–109	1
D10Rat20*	F: AGTGATTGCCATACCTGCCT R: GAAATGGCCAGGATAAACCA	Jacob et al. (1995)	HEX	NA	1
D20Rat46	F: GGCAAAACACCAATGCCTAT R: AAGTACTGAGTGGGCTGCGT	Jacob et al. (1995)	TAMRA	145–185	1
D5Rat83^#^	F: ACTTGGAAACAGGGAGATGG R: GGTCTTCAGGATGGCAATGT	Jacob et al. (1995)	HEX	163–196	1
D15Rat77^#^	F: ACAGAGGGAACCCATCACAG R: CATGTGGGGAAAGCATTACC	Jacob et al. (1995)	TAMRA	222–260	1
Rr107^#^	F: CTGACAAAGGCAGCCAGTG R: CATCTGGATGTCTGCAGGATG	Loiseau et al. (2008)	HEX	268–314	1
Rr22^#^	F: CCGTAAGTAGAAGCTGGTTGAG R: CTGGTCCTTCTGAGGCTCTCT	Loiseau et al. (2008)	HEX	344–355	1
Rr14^#^	F: CTGGCTGGGACAGTGGAG R: CGTCATCACTTCTCAGGACAG	Loiseau et al. (2008)	TAMRA	107–147	2
D16Rat81^#^	F: GGCCCACATGTGCATGTATA R: GAGCCTTAGCACAGTGGCTT	Jacob et al. (1995)	FAM	134–160	2
Rr67^#^	F: CATCCTGTGACCTTGAAGTG R: ACATGTAAGGCAGAGGATGG	Loiseau et al. (2008)	HEX	177–187	2
Rr21^#^	F: AGTCAGTGTGGAGCAGGCA R: GAGAAATTCAAACCTCAACTGC	Loiseau et al. (2008)	HEX	209–234	2
D11Mgh5^#^	F: CAGCTCTAATTCCAGAAAGGTTT R: GAATCGATTGACAGATGTCTGTG	Jacob et al. (1995)	FAM	243–292	2
Rr93*	F: GAAAGATCATTTCCTGGACC R: GGAGCTGGTTCTCTACATCC	Loiseau et al. (2008)	TAMRA	NA	2
Rr114*	F: GCTGTGGCTAGAATCCAAGG R: ATGAGGCCTGTGGACGGTA	Loiseau et al. (2008)	TAMRA	NA	2
D2Rat234*	F: ATATTCAAGCTGGCTTCCCC R: GTAGAGCAAGATGGGGTGGA	Jacob et al. (1995)	FAM	NA	3
D7Rat13^#^	F: GACTTCTGCTACACGCCACA R: CAGCCCTAGAAGGAAATGCA	Jacob et al. (1995)	HEX	150–189	3
D19Mit2*	F: AAGGTTGGCAGTTTCCCAG R: ACCATTTATGTGCCCAGATG	Jacob et al. (1995)	FAM	NA	3
Rr17^#^	F: CGTGTGGCATAGGTGAAGG R: TGCAGGAAACTGGTAGGACA	Loiseau et al. (2008)	TAMRA	202–220	3
D18Rat96^#^	F: GCAGATCTCTCCTCCACAGC R: TGGACATCCTCAATGGACCT	Jacob et al. (1995)	HEX	225–244	3
Rr54^#^	F: AGCCACTGCGACAGAAAGC R: CATTAGCAAGCCTTCCTGGAG	Loiseau et al. (2008)	FAM	311–338	3

*Indicates loci subsequently excluded from analysis due to high rate of missing data and/or inconsistent amplification

^#^Indicates the optimal set of 14

### Data analyses

MtDNA D-loop sequences were trimmed and aligned using Geneious 11.1.4 (Kearse et al. 2012). These sequences were used to confirm species identity of each sample by comparison to published, homologous sequences using NCBI BLAST (Basic Local Alignment Search Tool; https://blast.ncbi.nlm.nih.gov/Blast.cgi). Aligned DNA sequences were trimmed, and a dataset was generated comprising one example of each haplotype using DnaSP 6.12.03 (Rozas et al. 2017). Haplotype diversity (Hd) and nucleotide diversity (π) were calculated in DnaSP 6.12.03, and we inferred a median‐joining network (Bandelt et al. 1999) using PopART (Leigh and Bryant 2015).

Microsatellite genotypes from ship rat specimens were scored using the Microsat plugin in Geneious. Genetic parameters for each population and each locus were calculated with Fstat 2.9.4 (Goudet 1995) and the R (R Core Team 2021) package PopGenReport (Adamack and Gruber 2014). To check for the presence of null alleles, Micro-Checker 2.2.3 (Van Oosterhout et al. 2004) was applied with 3,000 randomisations and the Bonferroni adjusted 95% confidence interval. A linkage disequilibrium test was applied to a single large population sample using Arlequin 3.5.2.2 (Excoffier and Lischer 2010) with the Markov chain steps and dememorization steps as 1,000,000 each and p-value as 0.003. Hardy–Weinberg exact tests were implemented in Fstat 2.9.4 (Raymond 1995) with 21,000 randomisations, and via GENEPOP on the Web (Rousset 2008, https://genepop.curtin.edu.au/) with the Markov chain parameters (dememorization, batches and iteration per batch) set to 10,000 iterations. During this exploratory process, five loci were removed from the dataset due to high frequencies of missing data and/or lack of allelic variation.

Pairwise F_ST_ values among population samples were estimated to assess population subdivision, using Arlequin 3.5.2.2 with 99,999 permutations and *p* values calculated with a significance level of 0.05 and a Bonferroni correction for multiple tests (28 tests: 0.05/28 = 0.00178). For populations at equilibrium statistically significant departures of pairwise F_ST_ values from zero indicate restricted gene flow between them. Average numbers of pairwise differences between populations (π_XY_) and within populations (π_X_) were estimated using Arlequin 3.5.2.2 with the same settings as F_ST_ estimation. A principal component analysis (PCA) was implemented in R 4.0 with the adegenet package 2.1.4 (Jombart 2008).

To estimate population structure by naïvely assigning individuals to clusters based on their genotypes, we used Bayesian model‐based analyses implemented in STRUCTURE 2.3.4 (Pritchard et al. 2000). An admixture model was applied with a burnin of 100,000 MCMC generations and 100,000 MCMC generations for analysis. Twenty iterations of each run were generated with the number of hypothetical populations/clusters (K) set to 1–8. Resulting simulations were then processed in STRUCTURE HARVESTER (Earl 2012) and the K-value best fitting the dataset was estimated by ΔK (Evanno et al. 2005). Subsequently, CLUMPP 1.1.2 (Jakobsson and Rosenberg 2007) and DISTRUCT 1.1 (Rosenberg 2004) were used to compile results for the optimal K and visualisation.

We used the R package GenePlot (McMillan and Fewster 2017) to assess the degree of similarity among individual microsatellite genotypes (14 loci) from population samples. In doing so GenePlot performs a genetic assignment test to determine the most likely source population of an individual.

## Results

### Mitochondrial haplotypes

Partial D-loop DNA sequences were used to confirm the identity of 243 samples. Among the 195 rodents studied from Picton, 193 were confirmed to be ship rats, and two tails were found to have come from house mice (*Mus musculus*) and excluded from further analysis. None of the samples were Norway rats. Similarly, 48 rat samples from North Island were confirmed to be ship rats and no Norway rats were sampled.

**Fig. 2:**
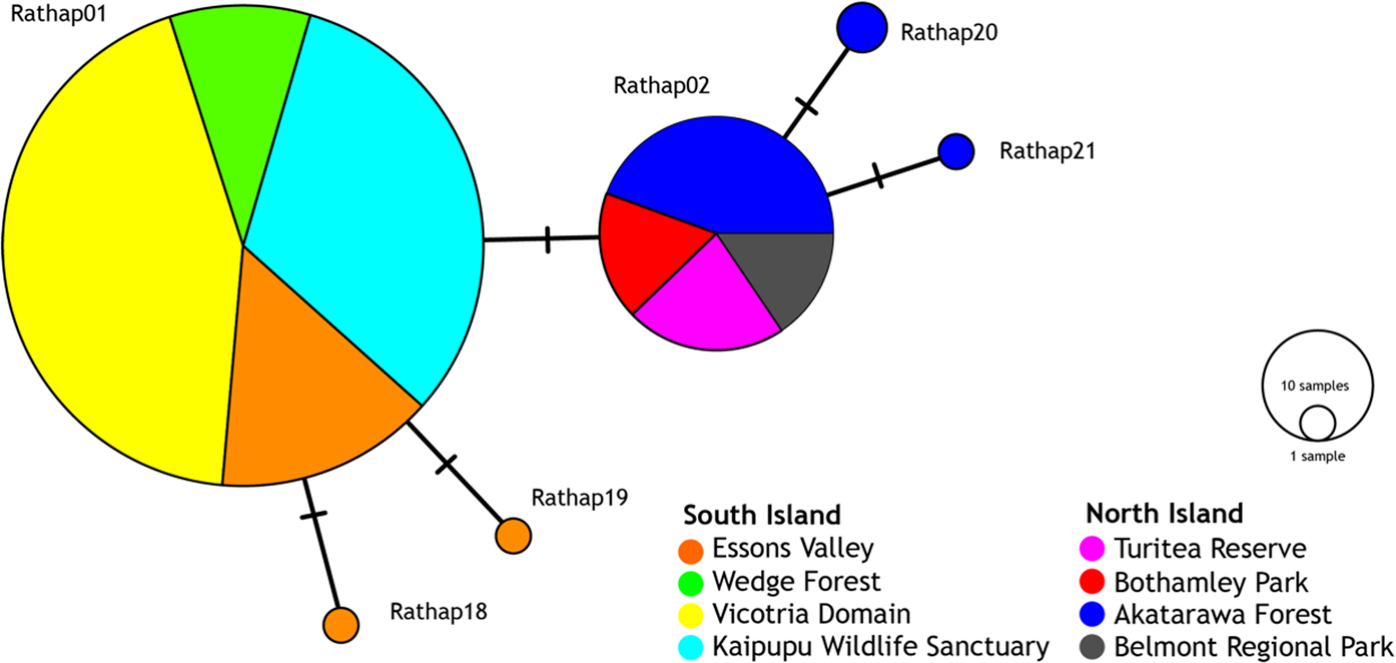
Median-Joinning network of six D-loop haplotypes (585 bp) recorded in ship rats (*Rattus rattus*) ship rats from North Island and South Island New Zealand

A total of 240 ship rat specimens resolved six haplotypes in a 585 bp segment of D-loop. These six haplotypes were differentiated by four (4/585) nucleotides (Fig. 2, See Supplementary Table S2). Two previously reported haplotypes (Russell et al. 2019) dominated our South Island (Rathap01) and North Island (Rathap02) population samples. No haplotypes were found in both North Island and South Island samples. Four haplotypes (Rathap18, Rathap19, Rathap20, Rathap21; Genbank accession numbers OM472144–OM472147) were confirmed as novel by comparison to published data (See Supplementary Tables S3 and S4, and Supplementary Fig. S1). These new haplotypes were recorded in five individuals, and differed by only one base from the locally common haplotype, either Rathap01 or Rathap02 (Fig. 2. See Supplementary Table S2). Overall haplotype diversity (*Hd*) for these six haplotypes was 0.033 and nucleotide diversity per site (π) was 0.00015.

### Microsatellite genotyping

Microsatellite genotypes were obtained for 133 ship rat specimens whose identity was first confirmed by mtDNA D-loop sequencing. These comprised 85 individuals from Picton (South Island) and 48 from North Island. For each population sample and each of 15 microsatellite loci (Table 1) we tested for the presence of null alleles and deviations of allele frequency from random using the Hardy–Weinberg exact test implemented with Micro-Checker 2.2.3, Fstat 2.9.4, and GENEPOP on the web. For only one locus, D20Rat46, did we find consistent evidence from all tests for null alleles and deviations from expected Hardy–Weinberg proportions. Subsequent analyses were therefore applied separately to data with 15 loci and 14 loci, but the presence of locus D20Rat46 did not result in any significant differences. Nevertheless, we note that chromatogram peaks for D20Rat46 alleles sometimes overlapped with those of D5Rat83 resulting in missed allele calls. This was confirmed by reamplification with the group 1 multiplex without D5Rat83, so we conservatively removed D20Rat46 data and here report the result of 133 samples with 14 loci (Table 2). Linkage disequilibrium tests were applied to three well-sampled populations; Kaipupu (n = 21), Victoria Domain (n = 26) and Akatarawa Forest (n = 23), revealing no consistent signal of linkage among these 14 loci (Table 1). These 14 loci had 2–19 alleles per sample, a total of 129 alleles and Hs ranging from 0.045 to 0.89 (Supplementary Table S5).

**Table 2:** Summary of nuclear genetic variation among New Zealand ship rats (*Rattus rattus*) sampled at eight locations with 14 microsatellite loci

Location	N_s_	N_a_	A_r_	PA	H_s_	H_o_	F_IS_
Kaipupu	21	50	3.09	4	0.516	0.556	− 0.077
Wedge	16	60	3.44	0	0.539	0.581	− 0.078
Victoria	26	69	3.61	1	0.556	0.533	0.040
Essons	22	66	3.63	2	0.558	0.562	− 0.007
Akatarawa	23	84	4.21	9	0.642	0.646	− 0.007
Bothamley	8	49	3.41	1	0.560	0.583	− 0.040
Turitea	10	53	3.49	4	0.599	0.550	0.082
Belmont	7	49	3.50	3	0.600	0.674	− 0.122

Number of samples (N_s_), number of alleles (N_a_), mean allelic richness (A_r_), private alleles (PA), mean gene diversity per population (H_s_) and F_IS_ values from PopGenReport and Fstat 2.9.4. Observed heterozygosity (H_o_) was calculated as H_S _− (F_IS_ × H_S_)

The highest genetic diversity was detected in the rat sample from Akatarawa Forest (n = 23; H_s_ = 0.642; Table 2), and the lowest in the sample from Kaipupu (n = 21; Hs = 0.516; Table 2). The Kaipupu sample had fewer alleles than any other sample but four alleles were detected only at Kaipupu (Table 2) and lowest π_X_ (Table 3). Estimates of inbreeding coefficient (F_IS_) provided no evidence of inbreeding within our sampled populations (six were negative and two < 0.1; Table 2). Estimates of population differentiation (F_ST_) for all pairwise population sample comparisons were significantly greater than zero, with the highest estimate of F_ST_ between North and South Island population samples (Table 3). Among the South Island population samples Kaipupu was moderately differentiated from the surrounding samples (pairwise F_ST_ estimates > 0.05) while rats from Wedge Forest were not significantly different from those caught at Victoria Domain and Essons Valley (Table 3). Highest pairwise F_ST_ and π_XY_ were those between Kaipupu sample and the population samples from North Island sites.

**Table 3:** Genetic differentiation among eight population samples of ship rat (*Rattus rattus*) from central New Zealand using 14 microsatellite loci

	Kaipupu	Wedge	Victoria	Essons	Akatarawa	Bothamley	Turitea	Belmont
Kaipupu	**7.19**	8.09***	8.01***	8.09***	11.68***	11.69***	11.67***	12.02***
Wedge	0.088***	**7.56**	7.83	7.83	11.46***	11.40***	11.29***	11.94***
Victoria	0.066***	0.021**	**7.77**	8.02**	11.60***	11.55***	11.63***	12.24***
Essons	0.072***	0.019*	0.028***	**7.81**	11.67***	11.57***	11.59***	12.14***
Akatarawa	0.306***	0.275***	0.279***	0.280***	**8.98**	8.82	9.19*	9.30*
Bothamley	0.373***	0.338***	0.335***	0.335***	0.058***	**7.54**	8.57**	8.77***
Turitea	0.341***	0.299***	0.311***	0.306***	0.055***	0.072***	**8.35**	9.07***
Belmont	0.360***	0.335***	0.343***	0.335***	0.058***	0.087**	0.072***	**8.48**

Pairwise F_ST_ (below diagonal), pairwise π_XY_ (above diagonal) and within population π_X_ (diagonal elements, bold). Grey cells indicate statistically significant pairwise F_ST_ and π_XY_ after Bonferroni correction

Significantly greater than zero, based on 99,999 permutations: *p < 0.05; **p < 0.01; ***p < 0.001

Applying Bonferroni correct for multiple tests only *p* < 0.001 are considered ‘significant’

**Fig. 3:**
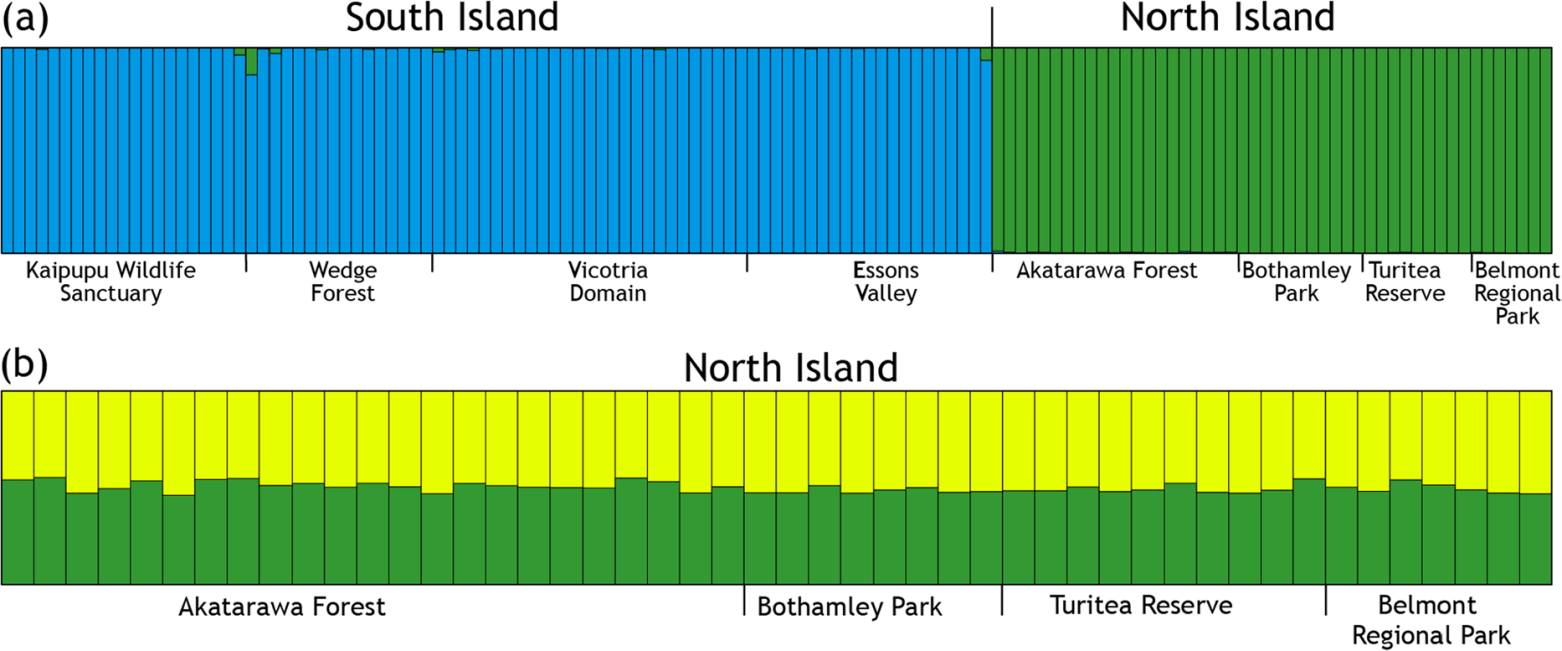
Genotypes of ship rats (*Rattus rattus*) reveals little genetic structure in southern North Island New Zealand, but distinguishes North Island and South Island samples (14 microsatellite loci). Eight population samples (North Island and South Island, New Zealand), K = 2 (**a**); Four population samples from southern North Island, K = 2 (**b**)

**Fig. 4:**
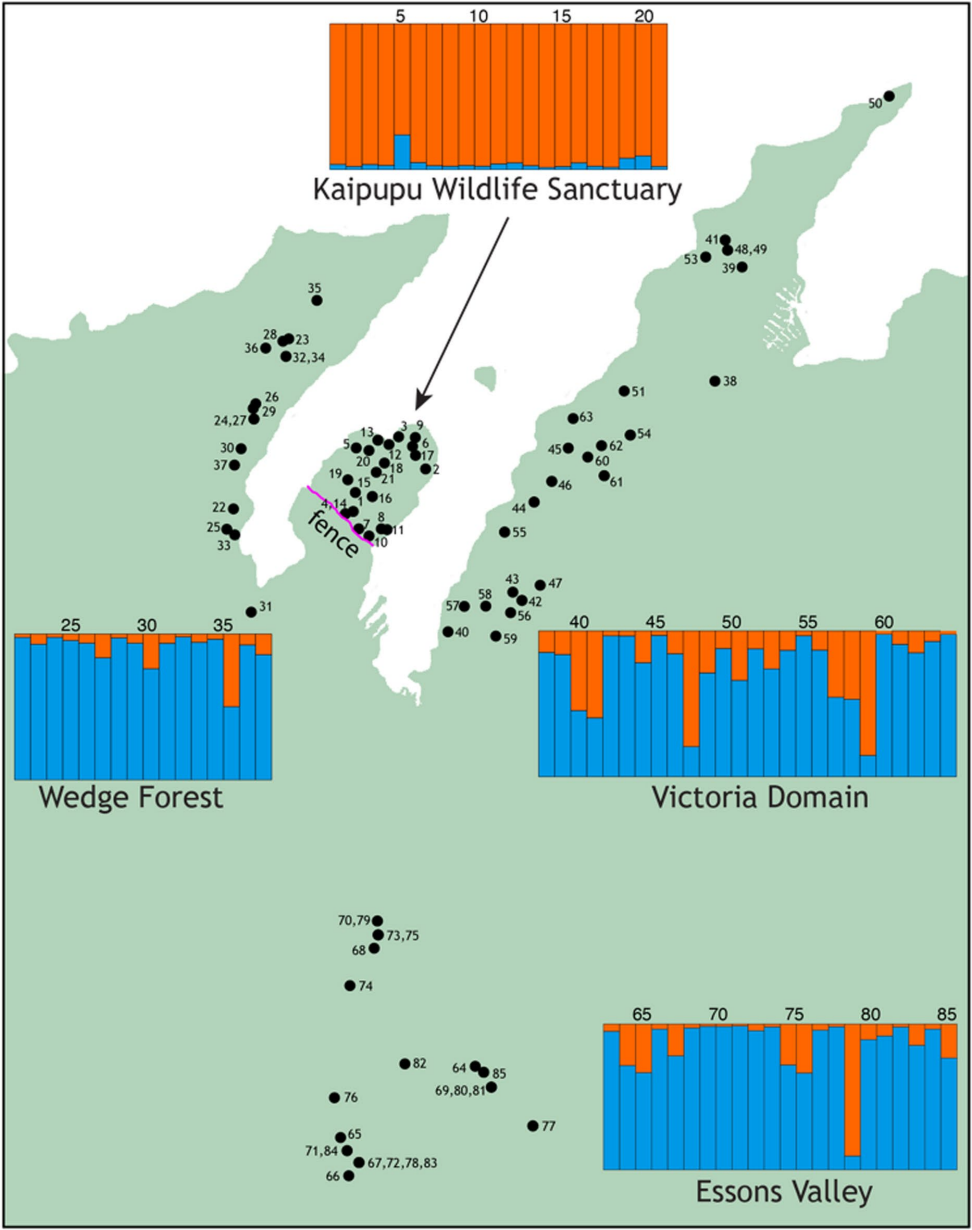
Population genetic structure of ship rats (*Rattus rattus*) around Picton, northern South Island, New Zealand. Locations for each rat trap and stacked bar plot showing assignment probabilities for each of 85 rats genotyped for 14 microsatellite loci (K = 2)

Applying a naïve Bayesian clustering approach to the genotypes of 133 rats with 14 loci revealed two clusters (K = 2, Fig. 3a. See also Supplementary Fig. S2). As predicted by F_ST_, rats in our sample from the North Island and the South Island were assigned to separate clusters. When data were partitioned by island, further evidence of population structure was apparent. Analysis of 48 rat genotypes from the four North Island population samples were assigned to two genetic clusters (K = 2, Supplementary Fig. S2) according to ΔK, but examination of assignment probabilities showed no evidence of two clusters among this set of individuals (effectively K = 1, Fig. 3b). In contrast, analysis of 85 rat genotypes from the Picton area clearly supported two genetic clusters; with few exceptions rats from Kaipupu represented a distinct genotype cluster compared to rats sampled from three adjacent areas (Fig. 4).

**Fig. 5:**
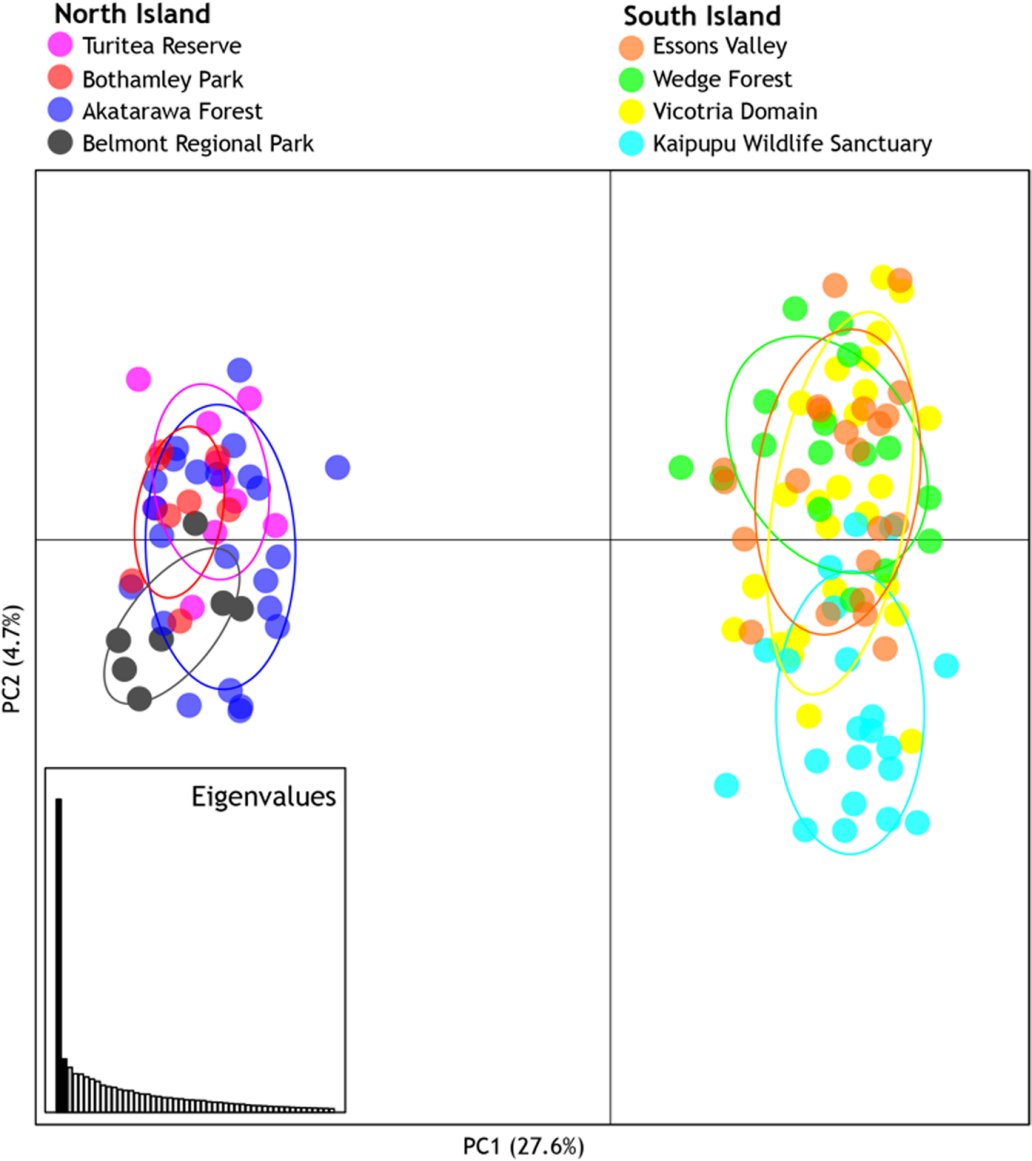
Ship rats (*Rattus rattus*) in North Island and South Island New Zealand are genetically differentiated. Principal component analysis of genotypes from 14 microsatellite loci from 133 rats where axes 1 and 2 explain 27.6% and 4.7% of the variance respectively

Although the hierarchical nature of genotypic clustering among the rats that we sampled was not apparent in the results of a single STRUCTURE analysis, application of principal component analysis was revealing in this respect. We found higher Euclidian distances between, rather than within, North Island and South Island samples, but the more subtle partitioning of Kaipupu rats from others in the Picton sample was also apparent (Fig. 5).

**Fig. 6:**
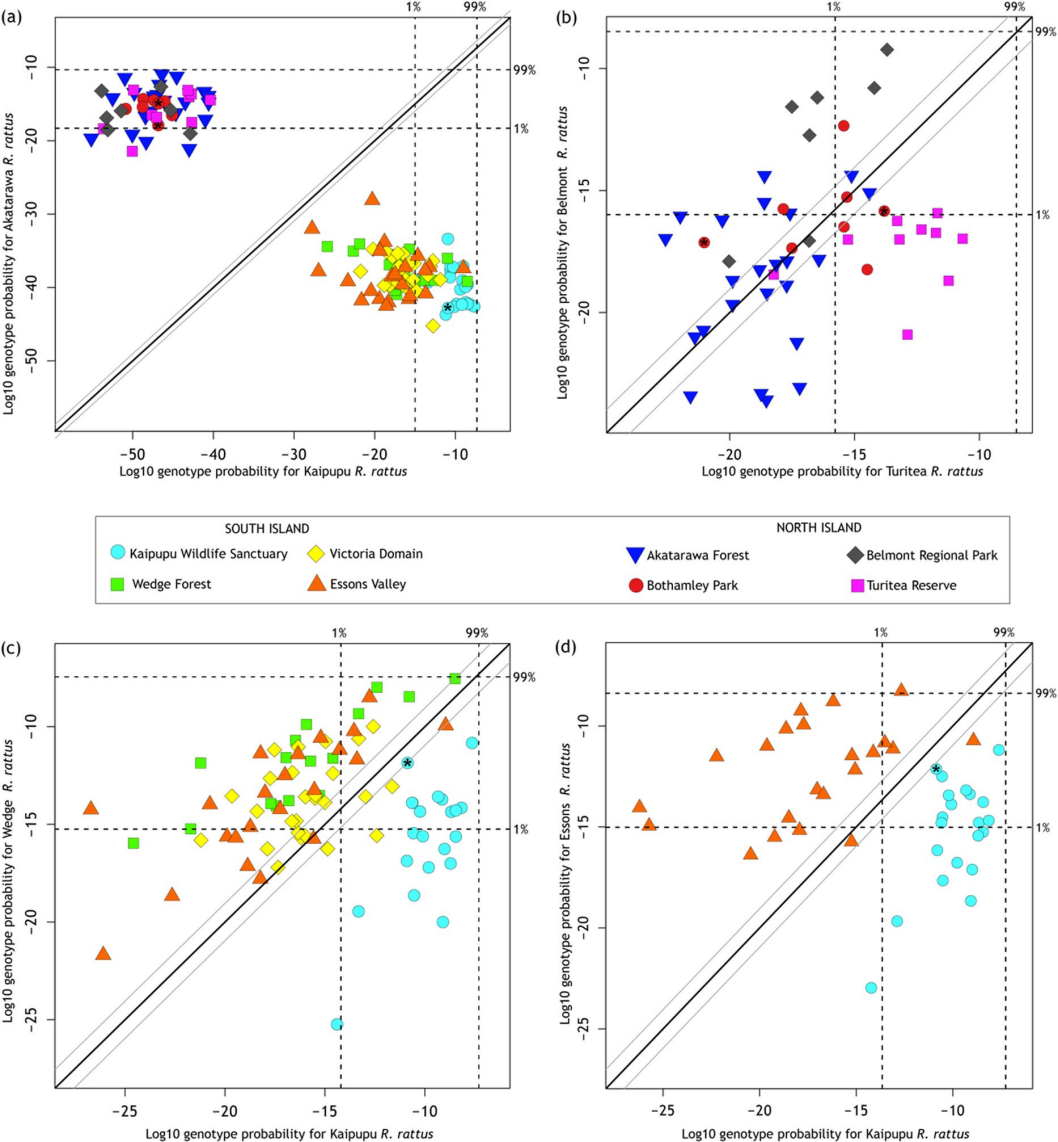
Assignment of origin for 133 New Zealand ship rats (*Rattus rattus*), using GenePlot (McMillan and Fewster 2017) analysis of genotypes from 14 microsatellite loci. Each point represents an individual rat. Rats from all eight location samples with Kaipupu Wildlife Sanctuary and Akatarawa Forest as reference populations (**a**); Rats from four North Island population samples with Turitea Reserve or Belmont Regional Park as reference populations (**b**); Rats from four South Island population samples around Picton with Kaipupu and Wedge Forest as reference populations (**c**); Rats from Kaipupu and Essons Valley with genetic assignment to each (**d**). Rats with asterisk had data missing at one or more loci with values calculated by quantile-approximation

Genetic assignment of individual rats to sampled populations also revealed that our rat samples were not genetically homogenous. All rats could be identified as originating from either North Island or South Island with high certainty (Fig. 6a). Genetic assignment of rats in North Island samples showed little evidence of differentiation even over 100 km, and with current sampling we have low confidence in correctly assigning rats to source locations at this scale (Supplemental Fig. S3 and Fig. 6b). Genetic assignment of individual rats to one of four (Fig. 6c) or one of two (Fig. 6d) populations around Picton revealed relatively high assignment probabilities, but the resolution was limited to assignment of rats to within Kaipupu, or outside the sanctuary (Fig. 6b, c). Pairwise assignment for Kaipupu rats and each of the three population samples around Picton identified three rats caught outside Kaipupu (in Essons Valley and Victoria Domain) that had a high probability of genetic assignment to the Kaipupu population (Fig. 6c).

## Discussion

Ship rats are reportedly more arboreal than Norway rats (Atkinson 1973) and are usually the more common of the two in New Zealand forests (Wilmshurst et al. 2021). Despite traps being set only on the ground, all rats captured for this study in southern North Island and northern South Island were confirmed to be ship rats by their mtDNA haplotype. Most of the sample sites were in forest, but even in urban Picton no Norway rats were encountered. The dynamics of the interaction between these two species is poorly understood but may be influenced by ground-hunting predators. For instance, in the absence of stoats, the two rat species coexist in New Zealand forest (Sturmer 1988), but mustelids are frequently captured in the Picton forests (reported by Picton Dawn Chorus) and southern North Island and feral cats abound (Pers. Obs).

Most of the ship rats in this study had mtDNA D-loop haplotypes already reported from New Zealand (Russell et al. 2019). The four new haplotypes each differed by only one nucleotide from the respective common local haplotype and have not previously been reported in any study of ship rats. It is most likely these novel haplotypes result from mutations in the large local rat population.

Our findings are consistent with previous observations that ship rats in most of South Island, New Zealand have a different mtDNA haplotype (Rathap01) from rats in the North Island (Rathap02; Russell et al. 2019). Both mtDNA sequences and nuclear microsatellite genotypes showed that our ship rat samples from North Island and South Island are genetically differentiated (Figs. 3, 5). Our dense sampling around Picton Harbour (northern South Island) is at the primary crossing point between the main islands, and the lack of evidence for gene flow indicates Cook Strait is a barrier to dispersal of rats despite daily inter-island shipping traffic.

As expected, multilocus microsatellite data provided higher resolution of genetic structure than the maternally inherited mtDNA haplotype variation and revealed population structure among ship rats sampled from locations no more than 9 kms apart. Notably, genetic differentiation of Kaipupu rats revealed this population sample as distinct from those in the adjacent forests that encircle Kaipupu with significant pairwise F_ST_ values after Bonferroni correction (Table 3). Population samples from Wedge Forest, Victoria Domain and Essons Valley (Fig. 1) were not genetically distinguishable from one another (Table 3), implying more gene flow among rats living in these areas. Genetic differentiation at these neutral loci is probably a result of genetic drift during a population bottleneck due to intense trapping within Kaipupu following fence construction. Our sample from Kaipupu had lower genetic diversity than other population samples in this study but had four private alleles and showed no statistical evidence of inbreeding.

The genetic distinction between rats in Kaipupu and those in nearby habitat (< 1 km) is readily apparent from naïve Bayesian clustering and principal component analysis of their genotypes (Figs. 4, 5). Assignment probabilities from the STRUCTURE analysis revealed that three individuals from Victoria Domain and Essons Valley had genotypes similar to the Kaipupu rats (Fig. 4, individuals #47, #58, #79), and genetic assignment with GenePlot (McMillan and Fewster 2017) also indicated these individuals had higher probability of originating in Kaipupu than other rats in the surrounding area (Fig. 6b). One individual from Essons Valley (Fig. 4, #79) consistently had a high genotype probability of being part of the Kaipupu population (Fig. 6c), while two individuals from Victoria Domain (Fig. 4, #47, #58) did not show evidence of a Kaipupu origin in pairwise genetic assignment analyses (Supplemental Fig. S3). Pairwise source population assignment for individual rats revealed that approximately half of the individuals from Kaipupu showed a reasonable fit to adjacent forest populations as well as to Kaipupu itself, while most individuals from adjacent forests had higher assignment probabilities for their own population (Fig. 6c, Supplemental Fig. S3). This all suggests our Kaipupu population sample is a genetic subset of the rats in the adjacent forest populations (McMillan and Fewster 2017), which can explain why some rats outside the reserve are so similar to the rats within the reserve and supports the idea that the Kaipupu population has experienced a population bottleneck. The genetic differentiation of Kaipupu rats from those in surrounding forest is consistent with the inference that the combination of predator-proof fence and water gap around Kaipupu restricts gene flow. It is possible that Kaipupu rats were already geographically isolated by town and sea before construction of the pest fence but the low density housing, gardens and near continuous scrub and forest provide ample habitat and dispersal corridors for rats (Fig. 1d). The same types of habitat exist among each of the sampled areas around Picton and demonstrate that urbanisation itself has not limited rat gene flow. Fine scale genetic structure among ship rats appears to be most strongly associated with isolation by water barriers not physical distance (Badou et al. 2021; Gilabert et al. 2007). In their study of ship rats using the same genetic markers as the present analysis Gatto-Almeida et al. (2020) found no significant genotypic difference among rats sampled on a narrow peninsula and those inland, but gaps (< 200 m) result in partitioning.

Given that a few rats sampled outside of Kaipupu are genetically similar to the population within the sanctuary, it is conceivable that individual rats are moving out from Kaipupu, however, it is equally likely that these genotypes detected in Victoria Domain and Essons Valley are related to rats that formed the founding population within Kaipupu. There is potential for rats to move in or out of Kaipupu on boats or by swimming the < 500 m water gap (King 2019; Russell et al. 2008), but our results reveal that ship rats are much more likely to disperse between nearby forest patches around Picton than into Kaipupu. Indeed we detected no barrier to gene flow among the forest sites outside Kaipupu even though they are separated by urban Picton, and this emphasises that suburbia does not constitute a dispersal barrier at this spatial scale.

In contrast to the genetic structure between Kaipupu rats and those in nearby forests, there was little genetic differentiation among the rats sampled from four more widely spaced forest locations in southern North Island. Although most pairwise F_ST_ values were greater than zero, pairwise genetic difference was similar to within sample variation (π_XY_, Table 3), and the four populations had overlapping variation in our principal component analysis. These North Island samples were undifferentiated in STRUCTURE analyses (Figs. 3, 5) and assignment of origin using GenePlot had low confidence. Sample size might limit these analyses but results suggest that these four rat populations experience as much gene flow as the Picton forest samples despite their greater geographic distances (> 100 km). Although the distance that individual ship rats can move is understood to be between several hundred metres and 1.5 km (Innes et al. 2011; Nathan et al. 2020), the population samples of ship rat in the North Island most likely represents a continuous distribution of this species through the Tararua Ranges and adjacent landscape (Fig. 1). In similar circumstances, genetic structure among ship rats was absent across 20 km^2^ of forest in Northland, New Zealand (Abdelkrim et al. 2010).

### Implications for pest management

The detection of genetic differentiation of the Kaipupu rat population from those in surrounding forest suggests there is a barrier to gene flow. Rats existed in the Kaipupu area before the predator fence was completed in 2010 and when initially established the existing predators were not eradicated. A shift in genotypic composition most likely reflects genetic drift during a population bottleneck associated with intense trapping within the fenced peninsula reserve, although it cannot be ruled-out that the peninsula alone generated this effect. In ideal circumstances, sampling of target pests before as well as after application of control measures (trapping, fencing, poison baiting etc.) would be useful in order to test for changes in allele frequency, but this is rarely achieved. Traditionally, wildlife population genetic analyses are used to infer population sizes and gene flow (Frankham 1996) without priors and this approach remains appropriate for pest species. Continued monitoring of population genotypes would nevertheless provide the opportunity to go beyond identifying population structure and enable potential drivers of population change to be assessed.

At Kaipupu, which is a relatively small area (40 ha), an eradication attempt using rodenticides (Russell and Broome 2016) could well be successful at relatively low cost. Maintenance of rat free status would undoubtedly require continued monitoring given the proximity of other terrestrial habitat and amount of water traffic, but resulting data would aid efforts to model eradication strategy and cost. Vagrant rats might occasionally be reaching Kaipupu but our data would suggest < 1 per generation, so an abrupt and intense suppression of rat density would favour detection of gene flow.

Although recent genomic methods allow the generation of large numbers of genetic markers (e.g. Combs et al. 2019) we found that 14 microsatellite loci were rich in allelic diversity. Microsatellite markers are readily and rapidly applied to temporal collections of tissue samples of various scale, form and condition (e.g. Vieira et al. 2016; Fox et al. 2019; Walker et al. 2020), and are informative in studies of rodent populations (e.g. Tollenaere et al. 2010; Varudkar and Ramakrshnan 2015; Guo et al. 2019; Gatto-Almeida et al. 2020). A key requirement for effective management of ecological pests is identification of changes in pest abundance, and intergenerational shifts in allele frequencies can show whether management actions result in biologically significant changes in population size and gene flow.

It is evident that ship rats exist in large, connected populations extending through regions of New Zealand such as the Tararua Ranges, so efforts at species eradication will need to identify and utilise any existing variance in rat density and gene flow. Large-scale dispersal barriers such as Cook Strait will be of value, but fine-scale subdivision of the rat population will be needed to decompose the eradication challenge into feasible parts.

A combination of landscape features can influence gene flow and the use of intense trapping can be sufficient to effect a change in the population structure of rats at local scales (as in Norway rats: Richardson et al. 2019). Arbitrary, intermittent localised trapping and poisoning, however, has little impact on the background abundance of these fecund, short-generation species (Clapperton et al. 2019). If management of rats and other pest mammals over an extensive, heterogenous environment is to be successful and eradication achieved, it is critical that we have a better understanding of fundamental pest population responses, and are equipped to apply appropriate tools during eradication efforts to monitor and counter such responses. Community groups (such as Picton Dawn Chorus) and other organisations have an important role in reducing competition with and predation of native species at a local level, but also an extremely valuable, under-utilised potential to contribute to the population genetic modelling that is required if pest species are to be eradicated over a large landscape (Tompkins 2018).

## Supplementary Information

Below is the link to the electronic supplementary material.

Supplementary file1 (DOCX 800 KB)

## Data Availability

The microsatellite dataset generated and analysed during the current study is available from the corresponding author on reasonable request. Mitochondrial DNA sequence data are available via GenBank (accessions numbers are reported in the text).
