# Supplementary material for: Genotypic detection of barriers to rat dispersal: Rattus rattus behind a peninsula predator-proof fence
Source: Biol Invasions. 2023 Feb 6;25(6):1723–38. doi: 10.1007/s10530-023-03004-8 (PMC9900205; doi:10.1007/s10530-023-03004-8)

**Genotypic detection of barriers to rat dispersal: *Rattus rattus* behind a predator proof fence.**

Shogo Yarita, Mary Morgan-Richards, Steven A. Trewick

Supplementary Table S1

*Rattus* sampling

| Sample # | Date collected | Location | Latitude | Longitude | Areas | D-loop | cyt b | Species | Microsat. |
| --- | --- | --- | --- | --- | --- | --- | --- | --- | --- |
| 1 | 14/08/19 | 1881 | -41.268795 | 174.034071 | Victoria domain | ○ |  | *R. rattus* | ○ |
| 5 | 4/10/19 | ME1150 | -41.277103 | 174.003548 | Kaipupu Wildlife Sanctuary | ○ |  | *R. rattus* | ○ |
| 10 | 25/10/19 | 876 | -41.27694 | 173.993447 | Wedge forest | ○ |  | *R. rattus* | ○ |
| 13 | 25/10/19 | 882 | -41.266101 | 173.998115 | Wedge forest | ○ |  | *R. rattus* | ○ |
| 17 | 11/10/19 | ME0200 | -41.274731 | 174.008856 | Kaipupu Wildlife Sanctuary | ○ |  | *R. rattus* | ○ |
| 18 | 6/10/19 | R0390 | -41.275133 | 174.006014 | Kaipupu Wildlife Sanctuary | ○ |  | *R. rattus* |  |
| 20 | 18/08/19 | 457 | -41.285723 | 174.011346 | Victoria domain | ○ |  | *R. rattus* |  |
| 25 | 25/10/19 | 835 | -41.27121 | 173.995174 | Wedge forest | ○ |  | *R. rattus* | ○ |
| 26 | 19/08/19 | 1165 | -41.312459 | 174.013847 | Essons valley | ○ |  | *R. rattus* | ○ |
| 28 | 26/06/19 | 628 | -41.279906 | 174.015975 | Victoria domain | ○ |  | *R. rattus* |  |
| 31 | 6/10/19 | R0390 | -41.275133 | 174.006014 | Kaipupu Wildlife Sanctuary | ○ |  | *R. rattus* |  |
| 32 | 20/09/19 | 879 | -41.278236 | 173.992886 | Wedge forest | ○ |  | *R. rattus* | ○ |
| 34 | 18/05/18 | 224 | -41.3169982 | 174.0024868 | Essons valley | ○ | ○ | *R. rattus* | ○ |
| 35 | 20/10/18 | W0860 | -41.272764 | 174.0073822 | Kaipupu Wildlife Sanctuary | ○ |  | *R. rattus* | ○ |
| 38 | 27/07/19 | C1130 | -41.278569 | 174.004227 | Kaipupu Wildlife Sanctuary | ○ |  | *R. rattus* |  |
| 39 | 5/08/19 | 666 | -41.261526 | 174.036358 | Victoria domain | ○ |  | *R. rattus* | ○ |
| 42 | 3/11/19 | MW0050 | -41.277255 | 174.00289 | Kaipupu Wildlife Sanctuary | ○ |  | *R. rattus* | ○ |
| 44 | 18/05/18 | 220 | -41.3194342 | 174.0031753 | Essons valley | ○ |  | *R. rattus* | ○ |
| 46 | 22/06/18 | 243 | -41.3185899 | 174.0040346 | Essons valley | ○ |  | *R. rattus* | ○ |
| 50 | 18/08/19 | 743 | -41.28477 | 174.011552 | Victoria domain | ○ |  | *R. rattus* | ○ |
| 59 | 18/03/19 | ME0100 | -41.273926 | 174.009098 | Kaipupu Wildlife Sanctuary | ○ |  | *R. rattus* |  |
| 61 | 18/05/18 | GN33 | -41.275125 | 174.009114 | Kaipupu Wildlife Sanctuary | ○ |  | *R. rattus* |  |
| 62 | 15/04/18 | 555 | -41.259807 | 174.034932 | Victoria domain | ○ |  | *R. rattus* | ○ |
| 63 | 3/07/18 | MW0210 | -41.276125 | 174.001708 | Kaipupu Wildlife Sanctuary | ○ |  | *R. rattus* |  |
| 64 | 11/03/19 | ME0550 | -41.276537 | 174.007703 | Kaipupu Wildlife Sanctuary | ○ |  | *R. rattus* |  |
| 66 | 27/04/18 | GN22 | -41.273426 | 174.00359 | Kaipupu Wildlife Sanctuary | ○ |  | *R. rattus* | ○ |
| 67 | 22/12/17 | 343 | -41.3128386 | 174.0145648 | Essons valley | ○ | ○ | *R. rattus* |  |
| 68 | 6/04/18 | C38b | -41.277513 | 174.00715 | Kaipupu Wildlife Sanctuary | ○ |  | *R. rattus* |  |
| 70 | 18/03/19 | ME1150 | -41.277103 | 174.003548 | Kaipupu Wildlife Sanctuary | ○ |  | *R. rattus* |  |
| 71 | 22/06/18 | 230 | -41.3049367 | 174.0053194 | Essons valley | ○ |  | *R. rattus* | ○ |
| 73 | 22/12/17 | 297 | -41.3137895 | 174.0152085 | Essons valley | ○ |  | *R. rattus* | ○ |
| 74 | 24/08/18 | 292 | -41.3032022 | 174.005589 | Essons valley | ○ |  | *R. rattus* | ○ |
| 75 | 18/05/18 | 239 | -41.3178366 | 174.0030349 | Essons valley | ○ |  | *R. rattus* | ○ |
| 80 | 26/06/19 | 556 | -41.275183 | 174.020304 | Victoria domain | ○ |  | *R. rattus* |  |
| 83 | 6/04/19 | W1125 | -41.2729544 | 174.0085597 | Kaipupu Wildlife Sanctuary | ○ |  | *R. rattus* | ○ |
| 84 | 22/03/19 | C1130 | -41.278569 | 174.004227 | Kaipupu Wildlife Sanctuary | ○ |  | *R. rattus* | ○ |
| 85 | 31/03/18 | GN25 | -41.272558 | 174.005636 | Kaipupu Wildlife Sanctuary | ○ |  | *R. rattus* |  |
| 86 | 15/04/18 | D06 | -41.274466 | 174.005311 | Kaipupu Wildlife Sanctuary | ○ |  | *R. rattus* |  |
| 87 | 22/06/19 | C0950 | -41.27821 | 174.005923 | Kaipupu Wildlife Sanctuary | ○ |  | *R. rattus* | ○ |
| 88 | 12/05/18 | DOC51 | -41.274724 | 174.001429 | Kaipupu Wildlife Sanctuary | ○ |  | *R. rattus* |  |
| 89 | 17/07/19 | 1914 | -41.274994 | 174.000844 | Kaipupu Wildlife Sanctuary | ○ |  | *R. rattus* |  |
| 90 | 23/03/19 | ME0200 | -41.274731 | 174.008856 | Kaipupu Wildlife Sanctuary | ○ |  | *R. rattus* |  |
| 91 | 15/12/18 | W1050 | -41.2723894 | 174.0087918 | Kaipupu Wildlife Sanctuary | ○ |  | *R. rattus* | ○ |
| 92 | 3/07/18 | W0370 | -41.2735136 | 174.0037599 | Kaipupu Wildlife Sanctuary | ○ |  | *R. rattus* |  |
| 93 | 22/06/19 | C1080 | -41.278716 | 174.004798 | Kaipupu Wildlife Sanctuary | ○ |  | *R. rattus* | ○ |
| 94 | 17/07/19 | 1914 | -41.274994 | 174.000844 | Kaipupu Wildlife Sanctuary | ○ |  | *R. rattus* |  |
| 95 | 25/08/18 | R0050 | -41.275087 | 174.003096 | Kaipupu Wildlife Sanctuary | ○ |  | *R. rattus* |  |
| 96 | 22/03/19 | C0900 | -41.278263 | 174.006415 | Kaipupu Wildlife Sanctuary | ○ |  | *R. rattus* | ○ |
| 97 | 27/04/18 | GN26 | -41.273116 | 174.006377 | Kaipupu Wildlife Sanctuary | ○ |  | *R. rattus* | ○ |
| 98 | 24/04/18 | GN22 | -41.273426 | 174.00359 | Kaipupu Wildlife Sanctuary | ○ |  | *R. rattus* |  |
| 99 | 22/06/19 | C1155m | -41.278439 | 174.003983 | Kaipupu Wildlife Sanctuary | ○ |  | *R. rattus* |  |
| 100 | 12/05/18 | GN25 | -41.272558 | 174.005636 | Kaipupu Wildlife Sanctuary | ○ |  | *R. rattus* | ○ |
| 101 | 25/05/19 | C0300 | -41.274396 | 174.00966 | Kaipupu Wildlife Sanctuary | ○ |  | *R. rattus* |  |
| 102 | 19/04/19 | MW0950 | -41.274018 | 174.006189 | Kaipupu Wildlife Sanctuary | ○ |  | *R. rattus* |  |
| 103 | 13/05/19 | QE0600 | -41.277762 | 174.005319 | Kaipupu Wildlife Sanctuary | ○ |  | *R. rattus* |  |
| 104 | 28/04/19 | R0540 | -41.276151 | 174.005169 | Kaipupu Wildlife Sanctuary | ○ |  | *R. rattus* |  |
| 105 | 25/08/18 | R0440 | -41.275562 | 174.006003 | Kaipupu Wildlife Sanctuary | ○ |  | *R. rattus* |  |
| 106 | 2/08/19 | MW0050 | -41.277255 | 174.00289 | Kaipupu Wildlife Sanctuary | ○ |  | *R. rattus* | ○ |
| 107 | 12/04/19 | ME0250 | -41.27473 | 174.008339 | Kaipupu Wildlife Sanctuary | ○ |  | *R. rattus* |  |
| 108 | 1/07/19 | QE0350 | -41.276356 | 174.006528 | Kaipupu Wildlife Sanctuary | ○ |  | *R. rattus* |  |
| 109 | 6/01/19 | W0410 | -41.2730543 | 174.0038068 | Kaipupu Wildlife Sanctuary | ○ |  | *R. rattus* |  |
| 110 | 10/03/19 | R0640 | -41.276006 | 174.004341 | Kaipupu Wildlife Sanctuary | ○ |  | *R. rattus* | ○ |
| 111 | 13/05/19 | QE0700 | -41.277523 | 174.004742 | Kaipupu Wildlife Sanctuary | ○ |  | *R. rattus* |  |
| 112 | 19/04/19 | ME0200 | -41.274731 | 174.008856 | Kaipupu Wildlife Sanctuary | ○ |  | *R. rattus* |  |
| 113 | 10/03/19 | R0540 | -41.276151 | 174.005169 | Kaipupu Wildlife Sanctuary | ○ | ○ | *R. rattus* | ○ |
| 114 | 12/04/19 | ME0700 | -41.277112 | 174.00644 | Kaipupu Wildlife Sanctuary | ○ |  | *R. rattus* |  |
| 115 | 10/08/18 | W0250 | -41.2734418 | 174.0026117 | Kaipupu Wildlife Sanctuary | ○ |  | *R. rattus* |  |
| 116 | 2/08/19 | MW0050 | -41.277255 | 174.00289 | Kaipupu Wildlife Sanctuary | ○ |  | *R. rattus* |  |
| 117 | 19/04/19 | W0805 | -41.2730875 | 174.0069513 | Kaipupu Wildlife Sanctuary | ○ |  | *R. rattus* |  |
| 119 | 15/09/18 | R0700 | -41.275897 | 174.00373 | Kaipupu Wildlife Sanctuary | ○ |  | *R. rattus* |  |
| 120 | 8/05/19 | ME0050 | -41.273529 | 174.008811 | Kaipupu Wildlife Sanctuary | ○ |  | *R. rattus* | ○ |
| 121 | 13/08/19 | QW0000 | -41.275558 | 174.001754 | Kaipupu Wildlife Sanctuary |  |  |  |  |
| 122 | 17/06/19 | R0750 | -41.275726 | 174.003254 | Kaipupu Wildlife Sanctuary | ○ |  | *R. rattus* |  |
| 123 | 22/06/19 | C0750 | -41.277376 | 174.007346 | Kaipupu Wildlife Sanctuary | ○ |  | *R. rattus* |  |
| 124 | 22/06/19 | R0640 | -41.276006 | 174.004341 | Kaipupu Wildlife Sanctuary | ○ |  | *R. rattus* |  |
| 125 | 22/06/19 | R0440 | -41.275562 | 174.006003 | Kaipupu Wildlife Sanctuary | ○ |  | *R. rattus* |  |
| 126 | 18/06/18 | 102 | -41.282771 | 174.0177874 | Victoria domain | ○ |  | *R.rattus* | ○ |
| 127 | 8/06/18 | 100 | -41.285591 | 174.014424 | Victoria domain |  |  |  |  |
| 128 | 27/05/18 | 243 | -41.3185899 | 174.0040346 | Essons valley | ○ |  | *R. rattus* | ○ |
| 129 | 27/05/18 | 229 | -41.3040852 | 174.0056576 | Essons valley | ○ |  | *R. rattus* | ○ |
| 130 | 27/05/18 | 235 | -41.3073199 | 174.0032775 | Essons valley | ○ |  | *R. rattus* | ○ |
| 131 | 25/05/18 | 167 | -41.282244 | 174.017015 | Victoria domain | ○ |  | *R. rattus* | ○ |
| 132 | 24/05/18 | 160 | -41.276504 | 174.018815 | Victoria domain | ○ |  | *R. rattus* | ○ |
| 133 | 20/04/18 | 629 | -41.280329 | 174.018019 | Victoria domain |  |  |  |  |
| 134 | 20/04/18 | 544 | -41.273065 | 174.021698 | Victoria domain | ○ |  | *R. rattus* | ○ |
| 135 | 20/05/18 | 244 | -41.2755286 | 174.0198361 | Victoria domain | ○ |  | *R. rattus* |  |
| 136 | 24/12/18 | 130 | -41.267499 | 174.025954 | Victoria domain | ○ |  | *R. rattus* |  |
| 137 | 20/05/18 | 138 | -41.278417 | 174.01633 | Victoria domain | ○ |  | *R. rattus* |  |
| 138 | 20/04/18 | 556 | -41.275183 | 174.020304 | Victoria domain | ○ |  | *R. rattus* | ○ |
| 139 | 27/10/17 | 101 | -41.280136 | 174.02017 | Victoria domain | ○ |  | *R. rattus* |  |
| 140 | 20/04/18 | 615 | -41.274822 | 174.024729 | Victoria domain | ○ |  | *R. rattus* |  |
| 141 | 20/05/18 | 172 | -41.275671 | 174.01896 | Victoria domain |  |  |  |  |
| 142 | 19/05/18 | 594 | -41.263472 | 174.032551 | Victoria domain |  |  |  |  |
| 143 | 20/04/18 | 575 | -41.269755 | 174.025652 | Victoria domain | ○ |  | *R. rattus* |  |
| 144 | 1/07/19 | QW0680 | -41.27456 | 174.006296 | Kaipupu Wildlife Sanctuary | ○ |  | *R. rattus* | ○ |
| 145 | 11/05/19 | R0090 | -41.275156 | 174.003591 | Kaipupu Wildlife Sanctuary | ○ |  | *R. rattus* | ○ |
| 146 | 19/05/18 | 587 | -41.266096 | 174.029985 | Victoria domain | ○ |  | *R. rattus* |  |
| 147 | 19/05/18 | 611 | -41.264031 | 174.03162 | Victoria domain |  |  |  |  |
| 148 | 1/07/19 | QW0450 | -41.273498 | 174.004878 | Kaipupu Wildlife Sanctuary | ○ |  | *R. rattus* | ○ |
| 149 | 6/01/19 | W0860 | -41.272764 | 174.0073822 | Kaipupu Wildlife Sanctuary | ○ |  | *R. rattus* |  |
| 150 | 11/07/19 | R0390 | -41.275133 | 174.006014 | Kaipupu Wildlife Sanctuary | ○ |  | *R. rattus* |  |
| 151 | 16/04/18 | 194 | -41.268519 | 174.028015 | Victoria domain |  |  |  |  |
| 152 | 28/04/18 | 370 | -41.257173 | 174.036836 | Victoria domain | ○ |  | *R. rattus* |  |
| 153 | 16/04/18 | 151 | -41.254631 | 174.042022 | Victoria domain | ○ |  | *R. rattus* |  |
| 154 | 28/04/18 | 163 | -41.253519 | 174.044281 | Victoria domain | ○ |  | *R. rattus* |  |
| 155 | 20/05/18 | 391 | -41.262572 | 174.031786 | Victoria domain |  |  |  |  |
| 156 | 16/04/18 | 163 | -41.253519 | 174.044281 | Victoria domain | ○ |  | *R. rattus* |  |
| 157 | 3/02/18 | 122 | -41.270545 | 174.022903 | Victoria domain | ○ |  | *R. rattus* |  |
| 158 | 4/05/18 | 76 | -41.281584 | 174.014201 | Victoria domain |  |  |  |  |
| 159 | 16/04/18 | 116 | -41.269998 | 174.025055 | Victoria domain |  |  |  |  |
| 160 | 6/05/18 | 148 | -41.25592 | 174.038737 | Victoria domain |  |  |  |  |
| 161 | 20/05/18 | 311 | -41.26595 | 174.027706 | Victoria domain |  |  |  |  |
| 162 | 14/09/18 | 229 | -41.3040852 | 174.0056576 | Essons valley | ○ |  | *R. rattus* | ○ |
| 163 | 1/04/18 | 560 | -41.259005 | 174.035448 | Victoria domain | ○ |  | *R. rattus* |  |
| 164 | 11/06/18 | 516 | -41.283739 | 174.015491 | Victoria domain |  |  |  |  |
| 165 | 29/07/18 | 231 | -41.3144617 | 174.0019665 | Essons valley | ○ |  | *R. rattus* | ○ |
| 166 | 2/11/18 | 280 | -41.3162559 | 174.0187108 | Essons valley | ○ |  | *R. rattus* | ○ |
| 167 | 13/07/18 | 280 | -41.3162559 | 174.0187108 | Essons valley | ○ |  | *R. rattus* |  |
| 168 | 15/12/17 | 86 | -41.281802 | 174.019327 | Victoria domain |  |  |  |  |
| 169 | 10/08/18 | 621 | -41.259365 | 174.034153 | Victoria domain | ○ |  | *R. rattus* |  |
| 170 | 20/07/18 | 165 | -41.267138 | 174.026251 | Victoria domain | ○ |  | *R. rattus* |  |
| 171 | 27/04/18 | 629 | -41.280329 | 174.018019 | Victoria domain | ○ |  | *R. rattus* | ○ |
| 172 | 20/02/18 | 219WR | -41.2744038 | 174.029974 | Victoria domain | ○ |  | *R. rattus* |  |
| 173 | 25/08/18 | 604 | -41.26844 | 174.028278 | Victoria domain |  |  |  |  |
| 174 | 6/05/18 | 164 | -41.26045 | 174.03514 | Victoria domain | ○ |  | *R. rattus* | ○ |
| 175 | 27/04/18 | 138 | -41.278417 | 174.01633 | Victoria domain | ○ |  | *R. rattus* |  |
| 176 | 6/05/18 | 628 | -41.279906 | 174.015975 | Victoria domain | ○ |  | *R. rattus* |  |
| 177 | 11/06/18 | 584 | -41.279505 | 174.016248 | Victoria domain | ○ |  | *R. rattus* |  |
| 178 | 11/06/18 | 243 | -41.3185899 | 174.0040346 | Essons valley | ○ |  | *R. rattus* | ○ |
| 179 | 25/05/18 | 182 | -41.2722344 | 174.0269297 | Victoria domain |  |  |  |  |
| 180 | 28/04/18 | 164 | -41.26045 | 174.03514 | Victoria domain | ○ |  | *R. rattus* | ○ |
| 181 | 21/04/18 | 138 | -41.278417 | 174.01633 | Victoria domain |  |  |  |  |
| 182 | 20/05/18 | 629 | -41.280329 | 174.018019 | Victoria domain | ○ |  | *Mus musculus* |  |
| 183 | 27/04/18 | 160 | -41.276504 | 174.018815 | Victoria domain | ○ |  | *R. rattus* |  |
| 184 | 14/05/18 | 226 | -41.276464 | 174.0205141 | Victoria domain | ○ |  | *R. rattus* |  |
| 185 | 6/07/18 | 567 | -41.274128 | 174.025409 | Victoria domain | ○ |  | *R. rattus* |  |
| 186 | 3/08/18 | 130 | -41.267499 | 174.025954 | Victoria domain |  |  |  |  |
| 187 | 26/03/18 | 148 | -41.25592 | 174.038737 | Victoria domain |  |  |  |  |
| 188 | 9/06/18 | 157 | -41.261416 | 174.034504 | Victoria domain | ○ |  | *R. rattus* |  |
| 189 | 16/04/18 | 166 | -41.266704 | 174.029282 | Victoria domain | ○ |  | *R. rattus* |  |
| 190 | 20/05/18 | 188 | -41.250636 | 174.048769 | Victoria domain | ○ |  | *R. rattus* | ○ |
| 191 | 27/04/18 | 182 | -41.2722344 | 174.0269297 | Victoria domain | ○ |  | *R. rattus* |  |
| 192 | 15/04/18 | 541 | -41.272911 | 174.024503 | Victoria domain |  |  |  |  |
| 193 | 1/04/18 | 609 | -41.270015 | 174.025575 | Victoria domain | ○ |  | *R. rattus* | ○ |
| 194 | 18/04/18 | 188 | -41.250636 | 174.048769 | Victoria domain |  |  |  |  |
| 195 | 30/03/18 | 154 | -41.280752 | 174.01468 | Victoria domain | ○ |  | *R. rattus* | ○ |
| 196 | 6/05/18 | 104 | -41.272615 | 174.026637 | Victoria domain | ○ |  | *R. rattus* |  |
| 197 | 6/05/18 | 175 | -41.27118 | 174.022105 | Victoria domain | ○ |  | *R. rattus* |  |
| 198 | 9/06/18 | 404 | -41.260885 | 174.033295 | Victoria domain | ○ |  | *R. rattus* | ○ |
| 200 | 26/03/18 | 163 | -41.253519 | 174.044281 | Victoria domain |  |  |  |  |
| 201 | 20/04/18 | 292 | -41.3032022 | 174.005589 | Essons valley | ○ |  | *R. rattus* | ○ |
| 202 | 26/03/18 | 166 | -41.266704 | 174.029282 | Victoria domain |  |  |  |  |
| 203 | 18/06/18 | 171 | -41.280211 | 174.013578 | Victoria domain | ○ |  | *R. rattus* |  |
| 204 | 28/07/18 | 297 | -41.3137895 | 174.0152085 | Essons valley | ○ |  | *R. rattus* | ○ |
| 205 | 22/06/18 | 182 | -41.2722344 | 174.0269297 | Victoria domain | ○ |  | *R. rattus* | ○ |
| 206 | 22/06/18 | 158 | -41.279367 | 174.013913 | Victoria domain |  |  |  |  |
| 207 | 18/06/18 | 117 | -41.276543 | 174.016798 | Victoria domain | ○ |  | *R. rattus* |  |
| 208 | 22/04/18 | 101 | -41.280136 | 174.02017 | Victoria domain | ○ |  | *R. rattus* |  |
| 209 | 22/06/18 | 136 | -41.277798 | 174.014922 | Victoria domain | ○ |  | *R. rattus* | ○ |
| 210 | 25/03/18 | 555 | -41.259807 | 174.034932 | Victoria domain | ○ |  | *R. rattus* |  |
| 211 | 27/04/18 | 512 | -41.283522 | 174.016064 | Victoria domain | ○ |  | *R. rattus* | ○ |
| 212 | 6/05/18 | 128 | -41.259263 | 174.035961 | Victoria domain | ○ |  | *R. rattus* |  |
| 213 | 4/05/18 | 62 | -41.278408 | 174.0209 | Victoria domain |  |  |  |  |
| 214 | 28/07/18 | 298 | -41.3150061 | 174.0168276 | Essons valley | ○ |  | *R. rattus* |  |
| 215 | 16/06/18 | 567 | -41.274128 | 174.025409 | Victoria domain |  |  |  |  |
| 216 | 26/03/18 | 142 | -41.268886 | 174.026842 | Victoria domain |  |  |  |  |
| 217 | 13/07/18 | 229 | -41.3040852 | 174.0056576 | Essons valley | ○ |  | *R. rattus* |  |
| 218 | 7/04/18 | 97 | -41.28315 | 174.012922 | Victoria domain | ○ |  | *R. rattus* | ○ |
| 219 | 19/05/18 | 574 | -41.274364 | 174.020779 | Victoria domain | ○ |  | *R. rattus* |  |
| 220 | 6/05/18 | 168 | -41.262245 | 174.03375 | Victoria domain |  |  |  |  |
| 221 | 16/06/18 | 541 | -41.272911 | 174.024503 | Victoria domain | ○ |  | *R. rattus* |  |
| 222 | 23/12/17 | 86 | -41.281802 | 174.019327 | Victoria domain |  |  |  |  |
| 223 | 11/03/18 | 297 | -41.3137895 | 174.0152085 | Essons valley | ○ |  | *R. rattus* | ○ |
| 224 | 7/04/18 | 154 | -41.280752 | 174.01468 | Victoria domain |  |  |  |  |
| 225 | 7/04/18 | 102 | -41.282771 | 174.0177874 | Victoria domain | ○ | ○ | *R. rattus* |  |
| 226 | 15/07/18 | 594 | -41.263472 | 174.032551 | Victoria domain | ○ |  | *R. rattus* |  |
| 227 | 14/05/19 | 7AC | -41.267729 | 174.0491706 | Victoria domain | ○ |  | *R. rattus* |  |
| 228 | 24/06/18 | 376 | -41.2831338 | 174.0147351 | Victoria domain | ○ |  | *R. rattus* | ○ |
| 229 | 17/02/19 | 896 | -41.27024 | 173.995326 | Wedge forest | ○ |  | *R. rattus* | ○ |
| 230 | 2/07/18 | 189 | -41.27536 | 174.017044 | Victoria domain | ○ |  | *R. rattus* |  |
| 231 | 6/07/18 | 154 | -41.280752 | 174.01468 | Victoria domain | ○ |  | *R. rattus* |  |
| 232 | 11/05/19 | 1186 | -41.312306 | 174.007915 | Essons valley | ○ |  | *R. rattus* | ○ |
| 233 | 14/09/18 | 618 | -41.261048 | 174.034964 | Victoria domain | ○ |  | *R. rattus* |  |
| 234 | 11/05/19 | 307 | -41.310292 | 174.0058315 | Essons valley | ○ |  | *R. rattus* |  |
| 235 | 15/09/18 | 68 | -41.2850425 | 174.0155867 | Victoria domain | ○ |  | *R. rattus* | ○ |
| 236 | 17/02/19 | 835 | -41.27121 | 173.995174 | Wedge forest | ○ |  | *R. rattus* | ○ |
| 237 | 15/03/19 | 844 | -41.266253 | 173.997618 | Wedge forest | ○ |  | *R. rattus* | ○ |
| 238 | 13/07/18 | 69 | -41.283552 | 174.016829 | Victoria domain | ○ |  | *R. rattus* |  |
| 239 | 22/07/19 | 1119 | -41.310518 | 174.005916 | Essons valley | ○ |  | *Mus musculus* |  |
| 240 | 24/06/18 | 91 | -41.288143 | 174.011277 | Victoria domain |  |  |  |  |
| 241 | 24/06/18 | 379 | -41.282327 | 174.018645 | Victoria domain |  |  |  |  |
| 242 | 15/03/19 | 839 | -41.270542 | 173.995114 | Wedge forest | ○ |  | *R. rattus* | ○ |
| 243 | 23/06/18 | 565 | -41.270259 | 174.026644 | Victoria domain |  |  |  |  |
| 244 | 22/03/19 | 229 | -41.3040852 | 174.0056576 | Essons valley | ○ |  | *R. rattus* |  |
| 245 | 23/06/18 | 333 | -41.292827 | 174.013906 | Essons valley | ○ |  | *R. rattus* |  |
| 246 | 26/04/19 | 825 | -41.273105 | 173.99409 | Wedge forest | ○ |  | *R. rattus* | ○ |
| 247 | 30/06/18 | 625 | -41.269429 | 174.026412 | Victoria domain | ○ |  | *R. rattus* |  |
| 248 | 7/07/18 | 117 | -41.276543 | 174.016798 | Victoria domain | ○ |  | *R. rattus* |  |
| 249 | 16/03/19 | 924 | -41.283517 | 173.994947 | Wedge forest | ○ |  | *R. rattus* | ○ |
| 250 | 28/12/18 | 897 | -41.278082 | 173.993365 | Wedge forest | ○ |  | *R. rattus* |  |
| 251 | 29/07/18 | 243 | -41.3185899 | 174.0040346 | Essons valley | ○ | ○ | *R. rattus* | ○ |
| 252 | 30/06/19 | 7AC | -41.267729 | 174.0491706 | Victoria domain | ○ |  | *R. rattus* |  |
| 253 | 2/07/18 | 191 | -41.277242 | 174.017042 | Victoria domain | ○ |  | *R. rattus* |  |
| 254 | 14/10/18 | 546 | -41.27154 | 174.022804 | Victoria domain |  |  |  |  |
| 255 | 22/06/18 | 107 | -41.286532 | 174.011604 | Victoria domain | ○ |  | *R. rattus* |  |
| 256 | 25/10/18 | 239 | -41.3178366 | 174.0030349 | Essons valley | ○ |  | *R. rattus* | ○ |
| 257 | 7/07/19 | 632 | -41.284497 | 174.016582 | Victoria domain |  |  |  |  |
| 259 | 15/03/19 | 885 | -41.26722 | 173.997877 | Wedge forest | ○ |  | *R. rattus* | ○ |
| 260 | 8/12/18 | 894 | -41.26386 | 174.000226 | Wedge forest | ○ |  | *R. rattus* |  |
| 261 | 30/06/18 | 611 | -41.264031 | 174.03162 | Victoria domain |  |  |  |  |
| 262 | 26/04/19 | 853 | -41.278592 | 173.993561 | Wedge forest | ○ |  | *R. rattus* | ○ |
| 263 | 1/07/18 | 480 | -41.2855222 | 174.0108016 | Victoria domain | ○ |  | *R. rattus* |  |
| 264 | 5/05/19 | 628 | -41.279906 | 174.015975 | Victoria domain |  |  |  |  |
| 265 | 10/09/18 | 525 | -41.273189 | 174.020817 | Victoria domain | ○ |  | *R. rattus* |  |
| 266 | 26/04/19 | 885 | -41.26722 | 173.997877 | Wedge forest | ○ |  | *R. rattus* | ○ |
| 267 | 1/10/18 | 280 | -41.3162559 | 174.0187108 | Essons valley | ○ |  | *R. rattus* |  |
| 268 | 8/12/18 | 871 | -41.263652 | 174.000491 | Wedge forest | ○ |  | *R. rattus* | ○ |
| 269 | 4/05/19 | 539 | -41.271083 | 174.028805 | Victoria domain |  |  |  |  |
| 270 | 6/07/18 | 62 | -41.278408 | 174.0209 | Victoria domain | ○ |  | *R. rattus* |  |
| 271 | 1/06/19 | 560 | -41.259005 | 174.035448 | Victoria domain | ○ |  | *R. rattus* |  |
| 272 | 6/07/18 | 138 | -41.278417 | 174.01633 | Victoria domain |  |  |  |  |
| 273 | 30/06/18 | 525 | -41.273189 | 174.020817 | Victoria domain | ○ |  | *R. rattus* |  |
| 274 | 7/07/19 | 86 | -41.281802 | 174.019327 | Victoria domain | ○ |  | *R. rattus* |  |
| 275 | 6/07/18 | 571 | -41.280286 | 174.016782 | Victoria domain | ○ |  | *R. rattus* |  |
| 276 | 5/04/19 | 81 | -41.273638 | 174.02334 | Victoria domain | ○ |  | *R. rattus* | ○ |
| 277 | 16/11/18 | 615 | -41.274822 | 174.024729 | Victoria domain | ○ |  | *R. rattus* | ○ |
| 278 | 22/07/19 | 343 | -41.3128386 | 174.0145648 | Essons valley | ○ |  | *R. rattus* | ○ |
| 279 | 5/04/19 | 92 | -41.27311 | 174.024295 | Victoria domain | ○ |  | *R. rattus* | ○ |
| 280 | 6/04/19 | 546 | -41.27154 | 174.022804 | Victoria domain | ○ |  | *R. rattus* | ○ |
| 281 | 4/05/19 | 567 | -41.274128 | 174.025409 | Victoria domain | ○ |  | *R. rattus* |  |
| 282 | 15/06/19 | 899 | -41.266694 | 173.996168 | Wedge forest | ○ |  | *R. rattus* | ○ |
| 283 | 18/01/19 | 836 | -41.274149 | 173.993531 | Wedge forest | ○ |  | *R. rattus* | ○ |
| 284 | 22/06/19 | R0390m | -41.2750953 | 174.0060459 | Kaipupu Wildlife Sanctuary | ○ |  | *R. rattus* | ○ |
| 285 | 16/02/20 | 576 | -41.2879684 | 174.0118837 | Victoria domain | ○ | ○ | *R. rattus* |  |

Supplementary Table S2

Nucleotide substitutions in mitochondrial D-loop sequences of *Rattus rattus* sampled either side of Cook Strait, New Zealand.

| **Haplotype** | **N** | **15495** | **15533** | **15635** | **15757** | **GenBank accession #** |
| --- | --- | --- | --- | --- | --- | --- |
| Rathap18 | 1 | A | G | C | C | OM472144 |
| Rathap19 | 1 | . | A | . | A | OM472145 |
| Rathap20 | 2 | G | A | . | T | OM472146 |
| Rathap21 | 1 | . | A | T | T | OM472147 |
| Rathap02 | 45 | . | A | . | T | KR559035 |
| Rathap01 | 190 | . | A | . | . | KR559034 |
| *Haplotype names follow Russell et al. (2019). *Position number is with reference to the published *R. rattus* mtDNA sequence EU273707 (Robins et al., 2008). | | | | | | |

Supplementary Table S3

131 haplotypes from partial (350 bp) mtDNA D-loop sequences from global *Rattus rattus* specimens. Asterisk * indicates uncertain sample size (< 3) which was counted as 1. Each area includes several country and islands: Madagascar – Madagascar, Grande Comore, Mayotte, Reunion Island, Europa Island, Juan De Nova Island and Grande Glorieuse; East Africa – Ethiopia, Tanzania, Mozambique and South Africa; West Africa – Senegal, Algeria and Tunisia; Europe – France, Greece, Italy, Lavezzi Island, Ouessant Island and Lundy Island; Middle East, Asia – India, Oman and Yemen; Oceania - Sideia Island, Samoa, Raiatea Island, Tahiti, Society Islands, Australia and New Zealand; North America – only New York; South America – Guadeloupe, Fernando de Noronha.­­

| Haplotype # | Number of samples in each area | | | | | | | | | total |
| --- | --- | --- | --- | --- | --- | --- | --- | --- | --- | --- |
|  | **Madagascar** | **East Africa** | **West Africa** | **Europe** | **Middle**  **East, Asia** | **Oceania** | **North**  **America** | **South America** | |  |
| 1 | 153* | 3 |  |  |  |  |  | |  | **156** |
| 2 | 4 |  |  |  |  |  |  | |  | **4** |
| 3 | 19 |  |  |  |  |  |  | |  | **19** |
| 4 | 5 |  |  |  |  |  |  | |  | **5** |
| 5 | 2* |  |  |  |  |  |  | |  | **2** |
| 6 | 1 |  |  |  |  |  |  | |  | **1** |
| 7 | 11 |  |  |  | 1 |  |  | |  | **12** |
| 8 | 6 |  |  |  |  |  |  | |  | **6** |
| 9 | 6 |  |  |  |  |  |  | |  | **6** |
| 10 | 2 |  |  |  |  |  |  | |  | **2** |
| 11 | 1 |  |  |  |  |  |  | |  | **1** |
| 12 | 1 |  |  |  |  |  |  | |  | **1** |
| 13 | 1 |  |  |  |  |  |  | |  | **1** |
| 14 |  | 2 | 35 | 44 |  | 437 | 1 | | 36* | **555** |
| 15 |  |  | 1 |  |  | 3 |  | |  | **4** |
| 16 |  |  |  |  |  |  |  | | 1* | **1** |
| 17 | 3 |  |  |  |  |  |  | |  | **3** |
| 18 | 2 |  |  |  |  |  |  | |  | **2** |
| 19 | 5 |  |  |  |  |  |  | |  | **5** |
| 20 | 3 |  |  |  |  |  |  | |  | **3** |
| 21 | 4 |  |  |  |  |  |  | |  | **4** |
| 22 | 2 |  |  |  |  |  |  | |  | **2** |
| 23 | 1 |  |  |  |  |  |  | |  | **1** |
| 24 | 1 |  |  |  |  |  |  | |  | **1** |
| 25 | 1 |  |  |  |  |  |  | |  | **1** |
| 26 | 2 |  |  |  |  |  |  | |  | **2** |
| 27 | 1 |  |  |  |  |  |  | |  | **1** |
| 28 | 1 |  |  |  |  |  |  | |  | **1** |
| 29 | 1 |  |  |  |  |  |  | |  | **1** |
| 30 | 1 |  |  |  |  |  |  | |  | **1** |
| 31 | 6 |  |  |  |  |  |  | |  | **6** |
| 32 | 1 |  |  |  |  |  |  | |  | **1** |
| 33 |  |  |  |  | 2 |  |  | |  | **2** |
| 34 |  |  |  |  | 1 |  |  | |  | **1** |
| 35 |  |  |  |  | 1 |  |  | |  | **1** |
| 36 |  | 1 |  |  | 1 |  |  | |  | **2** |
| 37 |  |  |  |  | 15 |  |  | |  | **15** |
| 38 |  |  |  |  | 3 |  |  | |  | **3** |
| 39 |  |  |  |  | 5 |  |  | |  | **5** |
| 40 |  |  |  |  | 2 |  |  | |  | **2** |
| 41 |  |  |  |  | 3 |  |  | |  | **3** |
| 42 |  |  |  |  | 1 |  |  | |  | **1** |
| 43 |  |  |  |  | 1 |  |  | |  | **1** |
| 44 |  |  |  |  | 1 |  |  | |  | **1** |
| 45 |  | 1 |  |  |  |  |  | |  | **1** |
| 46 |  | 1 |  |  |  |  |  | |  | **1** |
| 47 |  | 3 |  |  |  |  |  | |  | **3** |
| 48 | 26 | 4 |  |  | 1 |  |  | |  | **31** |
| 49 |  | 1 |  |  |  |  |  | |  | **1** |
| 50 |  | 1 |  |  |  |  |  | |  | **1** |
| 51 | 2 |  |  |  |  |  |  | |  | **2** |
| 52 | 1 |  |  |  |  |  |  | |  | **1** |
| 53 | 1 |  |  |  |  |  |  | |  | **1** |
| 54 | 7 |  |  |  |  | 1 |  | |  | **8** |
| 55 |  |  |  |  |  | 1 |  | |  | **1** |
| 56 | 1* |  |  |  |  |  |  | |  | **1** |
| 57 | 6 |  |  |  |  |  |  | |  | **6** |
| 58 | 1 |  |  |  |  |  |  | |  | **1** |
| 59 | 1 |  |  |  |  |  |  | |  | **1** |
| 60 | 1 |  |  |  |  |  |  | |  | **1** |
| 61 | 1 |  |  |  |  |  |  | |  | **1** |
| 62 | 1 |  |  |  |  |  |  | |  | **1** |
| 63 |  |  |  |  |  | 217 |  | | 23 | **240** |
| 64 |  |  |  | 1 |  | 16 |  | |  | **17** |
| 65 |  |  |  |  |  | 122 |  | |  | **122** |
| 66 |  |  |  |  |  | 1 |  | |  | **1** |
| 67 |  |  |  | 1 |  |  |  | |  | **1** |
| 68 |  |  |  | 2 |  |  |  | |  | **2** |
| 69 |  |  |  | 3 |  |  |  | |  | **3** |
| 70 |  |  | 1 |  |  |  |  | |  | **1** |
| 71 |  |  | 1 |  |  |  |  | |  | **1** |
| 72 |  |  |  | 1 |  |  |  | |  | **1** |
| 73 |  |  |  | 1 |  |  |  | |  | **1** |
| 74 |  |  | 1 |  |  |  |  | |  | **1** |
| 75 |  |  |  | 1 |  |  |  | |  | **1** |
| 76 |  |  |  | 1 |  |  |  | |  | **1** |
| 77 |  |  |  | 1 |  |  |  | |  | **1** |
| 78 |  |  |  | 1 |  |  |  | |  | **1** |
| 79 |  |  |  | 1 |  |  |  | |  | **1** |
| 80 |  |  |  |  |  | 27 |  | |  | **27** |
| 81 |  |  |  |  |  | 1 |  | |  | **1** |
| 82 |  |  |  |  |  | 3 |  | |  | **3** |
| 83 |  |  |  |  |  | 3 |  | |  | **3** |
| 84 |  |  |  |  |  | 1 |  | |  | **1** |
| 85 |  |  |  |  |  | 1 |  | |  | **1** |
| 86 |  |  |  |  |  | 1 |  | |  | **1** |
| 87 |  |  |  |  |  | 1 |  | |  | **1** |
| 88 |  |  |  |  |  | 1 |  | |  | **1** |
| 89 |  |  |  |  | 14 |  |  | |  | **14** |
| 90 |  |  |  |  | 1 |  |  | |  | **1** |
| 91 |  |  |  |  | 2 |  |  | |  | **2** |
| 92 |  |  |  |  | 1 |  |  | |  | **1** |
| 93 |  |  |  |  | 2 |  |  | |  | **2** |
| 94 |  |  |  |  | 5 |  |  | |  | **5** |
| 95 |  |  |  |  | 2 |  |  | |  | **2** |
| 96 |  |  |  |  | 2 |  |  | |  | **2** |
| 97 |  |  |  |  | 1 |  |  | |  | **1** |
| 98 |  |  |  |  | 1 |  |  | |  | **1** |
| 99 |  |  |  |  | 4 |  |  | |  | **4** |
| 100 |  |  |  |  | 8 |  |  | |  | **8** |
| 101 |  |  |  |  | 4 |  |  | |  | **4** |
| 102 |  |  |  |  | 1 |  |  | |  | **1** |
| 103 |  |  |  |  | 1 |  |  | |  | **1** |
| 104 |  |  |  |  | 2 |  |  | |  | **2** |
| 105 |  |  |  |  | 1 |  |  | |  | **1** |
| 106 |  |  |  |  | 5 |  |  | |  | **5** |
| 107 |  |  |  |  | 6 |  |  | |  | **6** |
| 108 |  |  |  |  | 2 |  |  | |  | **2** |
| 109 |  |  |  |  | 5 |  |  | |  | **5** |
| 110 |  |  |  |  | 10 |  |  | |  | **10** |
| 111 |  |  |  |  | 8 |  |  | |  | **8** |
| 112 |  |  |  |  | 2 |  |  | |  | **2** |
| 113 |  |  |  |  | 2 |  |  | |  | **2** |
| 114 |  |  |  |  | 6 |  |  | |  | **6** |
| 115 |  |  |  |  | 2 |  |  | |  | **2** |
| 116 |  |  |  |  | 4 |  |  | |  | **4** |
| 117 |  |  |  |  | 1 |  |  | |  | **1** |
| 118 |  |  |  |  | 11 |  |  | |  | **11** |
| 119 |  |  |  |  | 1 |  |  | |  | **1** |
| 120 |  |  |  |  | 3 |  |  | |  | **3** |
| 121 |  |  |  |  | 9 |  |  | |  | **9** |
| 122 |  |  |  |  | 4 |  |  | |  | **4** |
| 123 |  |  |  |  | 2 |  |  | |  | **2** |
| 124 |  |  |  |  | 1 |  |  | |  | **1** |
| 125 |  |  |  |  | 1 |  |  | |  | **1** |
| 126 |  |  |  |  | 6 |  |  | |  | **6** |
| 127 |  |  |  |  | 1 |  |  | |  | **1** |
| 128 |  |  |  |  | 3 |  |  | |  | **3** |
| 129 |  |  |  |  | 6 |  |  | |  | **6** |
| 130 |  |  |  |  |  | 2 |  | |  | **2** |
| 131 |  |  |  |  |  | 1 |  | |  | **1** |
| Total | **296** | **17** | **39** | **58** | **191** | **840** | **1** | | **60** | **1502** |

Supplementary Table S4 Details of 131 haplotypes detected among global *Rattus rattus* mtDNA D-Loop sequence data.

Note: Asterisk* indicates 4 haplotypes found from New Zealand rats in this study. The colour of highlight indicates the series of haplotype reported in each article.

| Haplotype | GenBank Accession Number | Haplotype code by the original authors | Locations |
| --- | --- | --- | --- |
| 1 | DQ009781 | H1 | Madagascar |
|  | GQ891588 | Haplotype 20 | Madagascar, Mozambique, Mayotte, Mozambique channel island |
|  | GQ891597 | Haplotype 29 | Mayotte |
|  | GQ891602 | Haplotype 34 | Madagascar |
| 2 | DQ009782 | H2 | Madagascar |
| 3 | DQ009783 | H3 | Madagascar |
|  | GQ891601 | Haplotype 33 | Madagascar |
|  | GQ891606 | Haplotype 38 | Madagascar |
| 4 | DQ009784 | H4 | Madagascar |
| 5 | DQ009785 | H5 | Madagascar |
|  | JF718276 | Haplotype 41 | Mozambique channel island |
| 6 | DQ009786 | H6 | Madagascar |
| 7 | DQ009787 | H7 | Madagascar |
|  | GQ891579 | Haplotype 11 | Oman |
| 8 | DQ009788 | H8 | Madagascar |
| 9 | DQ009789 | H9 | Madagascar |
|  | GQ891605 | Haplotype 37 | Madagascar |
| 10 | DQ009790 | H10 | Madagascar |
| 11 | DQ009791 | H11 | Madagascar |
| 12 | DQ009792 | H12 | Madagascar |
| 13 | DQ009793 | H13 | Madagascar |
| 14 | DQ009794 | HaMI | New York, Europe Islands, French Polynesia |
|  | EF186354 |  | French Polynesia |
|  | EF186355 |  | New Zealand |
|  | EF186357 |  | Papua New Guinea |
|  | EF186359 |  | French Polynesia |
|  | EF186360 |  | Samoa |
|  | EU273707 (NC_012374) |  | New Zealand |
|  | FJ897499 |  | Senegal |
|  | FJ897500 |  | Guadeloupe |
|  | GQ891608 | Haplotype 40 | South Africa |
|  | HQ588111 |  | French Polynesia |
|  | KR559034 | Rathap01 | New Zealand |
|  | LN554990 | Hap1 | Algeria, French, Greece, Italy, Tunisia |
|  | LN554991 | Hap2 | Tunisia |
|  | MN746385 |  | Fernande de Noronha (Brazil) |
|  | Picton1* |  | New Zealand |
| 15 | FJ897498 |  | Senegal |
|  | MH751499 | Rathap17 | New Zealand |
| 16 | FJ897501 |  | Guadeloupe |
| 17 | GQ891553 | H14 | Madagascar |
|  | GQ891600 | Haplotype 32 | Madagascar |
| 18 | GQ891554 | H15 | Madagascar |
| 19 | GQ891555 | H16 | Madagascar |
|  | GQ891599 | Haplotype 31 | Madagascar |
|  | JF718278 | Haplotype 43 | Mozambique channel Island |
|  | JF718279 | Haplotype 44 | Mozambique channel Island |
| 20 | GQ891556 | H17 | Madagascar |
|  | GQ891603 | Haplotype 35 | Madagascar |
| 21 | GQ891557 | H18 | Madagascar |
| 22 | GQ891558 | H19 | Madagascar |
| 23 | GQ891559 | H20 | Madagascar |
| 24 | GQ891560 | H21 | Madagascar |
| 25 | GQ891561 | H22 | Madagascar |
| 26 | GQ891562 | H23 | Madagascar |
|  | GQ891604 | Haplotype 36 | Madagascar |
| 27 | GQ891563 | H24 | Madagascar |
| 28 | GQ891564 | H25 | Madagascar |
| 29 | GQ891565 | H26 | Madagascar |
| 30 | GQ891566 | H27 | Madagascar |
| 31 | GQ891567 | H28 | Madagascar |
| 32 | GQ891568 | H29 | Madagascar |
| 33 | GQ891569 | Haplotype 1 | India |
|  | KJ603326 | BGR3 | India |
| 34 | GQ891570 | Haplotype 2 | India |
| 35 | GQ891571 | Haplotype 3 | India |
| 36 | GQ891572 | Haplotype 4 | India |
|  | GQ891587 | Haplotype 19 | Tanzania |
| 37 | GQ891573 | Haplotype 5 | India |
|  | KJ603344 | OTR1 | India |
|  | KJ603347 | OTR4 | India |
|  |  | OTR10 | India |
|  |  | OTR11 | India |
|  |  | OTR12 | India |
|  |  | OTR13 | India |
|  |  | OTR14 | India |
|  |  | OTR15 | India |
|  |  | OTR16 | India |
|  |  | OTR18 | India |
|  |  | OTR19 | India |
|  |  | OTR22 | India |
|  |  | OTR24 | India |
|  |  | OTR27 | India |
| 38 | GQ891574 | Haplotype 6 | Oman |
| 39 | GQ891575 | Haplotype 7 | Oman |
|  | KP159546 | KDR8 | India |
|  |  | KDR11 | India |
| 40 | GQ891576 | Haplotype 8 | Oman |
|  | KP159530 | ANR7 | India |
| 41 | GQ891577 | Haplotype 9 | Oman |
|  | GQ891578 | Haplotype 10 | Oman |
|  |  | AGR21 | India |
| 42 | GQ891580 | Haplotype 12 | Oman |
| 43 | GQ891581 | Haplotype 13 | Yemen |
| 44 | GQ891582 | Haplotype 14 | Yemen |
| 45 | GQ891583 | Haplotype 15 | Ethiopia |
| 46 | GQ891584 | Haplotype 16 | Ethiopia |
| 47 | GQ891585 | Haplotype 17 | Tanzania |
| 48 | GQ891586 | Haplotype 18 | Madagascar, Tanzania, Mozambique, Grande Comore |
|  | GQ891594 | Haplotype 26 | Grande Comore |
|  | GQ891595 | Haplotype 27 | Grande Comore |
|  | GQ891596 | Haplotype 28 | Grande Comore |
|  | KF282338 | Haplotype 46 | Madagascar |
|  | KF282339 | Haplotype 47 | Madagascar |
|  | KF282340 | H30 | Madagascar |
|  |  | THR21 | India |
| 49 | GQ891589 | Haplotype 21 | Mozambique |
| 50 | GQ891590 | Haplotype 22 | Mozambique |
| 51 | GQ891591 | Haplotype 23 | Grande Comore |
|  | GQ891592 | Haplotype 24 | Grande Comore |
| 52 | GQ891593 | Haplotype 25 | Grande Comore |
| 53 | GQ891598 | Haplotype 30 | Mayotte |
| 54 | GQ891607 | Haplotype 39 | Reunion Island |
|  | MH751496 | Rathap14 | New Zealand |
| 55 | HQ334447 |  | Australia |
| 56 | JF718277 | Haplotype 42 | Mozambique channel Island |
| 57 | KF282337 | Haplotype 45 | Madagascar |
|  | KF282341 | H31 | Madagascar |
| 58 | KF282342 | H32 | Madagascar |
| 59 | KF282343 | H33 | Madagascar |
| 60 | KF282344 | H34 | Madagascar |
| 61 | KF282345 | H35 | Madagascar |
| 62 | KF282346 | H36 | Madagascar |
| 63 | KR559035 | Rathap02 | New Zealand |
|  | MH751494 | Rathap12 | New Zealand |
|  | MN746386 |  | Fernando de Noronha (Brazil) |
| 64 | KR559036 | Rathap03 | New Zealand |
|  | LN555001 | Hap12 | Italy |
| 65 | KR559037 | Rathap07 | New Zealand |
|  | KR559039 | Rathap09 | New Zealand |
| 66 | KR559038 | Rathap08 | New Zealand |
| 67 | LN554992 | Hap3 | Italy |
| 68 | LN554993 | Hap4 | Italy |
| 69 | LN554994 | Hap5 | Italy |
| 70 | LN554995 | Hap6 | Tunisia |
| 71 | LN554996 | Hap7 | Algeria |
| 72 | LN554997 | Hap8 | French |
| 73 | LN554998 | Hap9 | Italy |
| 74 | LN554999 | Hap10 | Tunisia |
| 75 | LN555000 | Hap11 | Italy |
| 76 | LN555002 | Hap13 | Italy |
| 77 | LN555003 | Hap14 | Italy |
| 78 | LN555004 | Hap15 | Italy |
| 79 | LN555005 | Hap16 | Italy |
| 80 | MH751489 | Rathap04 | New Zealand |
| 81 | MH751490 | Rathap05 | New Zealand |
| 82 | MH751491 | Rathap06 | New Zealand |
| 83 | MH751492 | Rathap10 | New Zealand |
|  | MH751498 | Rathap16 | New Zealand |
| 84 | MH751493 | Rathap11 | New Zealand |
| 85 | MH751495 | Rathap13 | New Zealand |
| 86 | MH751497 | Rathap15 | New Zealand |
| 87 | OM472145 | **Rathap19*** | New Zealand |
| 88 | OM472144 | **Rathap18*** | New Zealand |
| 89 | KJ603317 | AGR1 | India |
|  | KP159524 | AGR2 | India |
|  | KP159525 | AGR3 | India |
|  | KP159526 | AGR4 | India |
|  | KP159527 | AGR5 | India |
|  | KP159528 | AGR6 | India |
|  |  | AGR12 | India |
|  |  | AGR13 | India |
|  |  | AGR14 | India |
|  |  | AGR15 | India |
|  |  | AGR16 | India |
|  |  | AGR17 | India |
|  |  | AGR18 | India |
|  |  | AGR19 | India |
| 90 | KP159529 | AGR7 | India |
| 91 |  | AGR8 | India |
|  |  | AGR10 | India |
| 92 |  | AGR9 | India |
| 93 |  | AGR11 | India |
|  |  | AGR20 | India |
| 94 | KJ603318 | ANR1 | India |
|  | KJ603321 | ANR4 | India |
|  | KJ603323 | ANR6 | India |
|  | KP159531 | ANR8 | India |
|  |  | ANR22 | India |
| 95 | KJ603319 | ANR2 | India |
|  |  | ANR23 | India |
| 96 | KJ603320 | ANR3 | India |
|  |  | ANR18 | India |
| 97 | KJ603322 | ANR5 | India |
| 98 | KP159532 | ANR9 | India |
| 99 | KP159533 | ANR10 | India |
|  | KP159539 | ANR16 | India |
|  |  | ANR20 | India |
|  |  | ANR29 | India |
| 100 | KP159534 | ANR11 | India |
|  | KP159536 | ANR13 | India |
|  | KP159538 | ANR15 | India |
|  |  | ANR17 | India |
|  |  | ANR19 | India |
|  |  | ANR21 | India |
|  |  | ANR24 | India |
|  |  | ANR26 | India |
| 101 | KP159535 | ANR12 | India |
|  |  | ANR28 | India |
|  |  | ANR30 | India |
|  |  | ANR31 | India |
| 102 | KP159537 | ANR14 | India |
| 103 |  | ANR25 | India |
| 104 |  | ANR27 | India |
|  | KJ603351 | THR4 | India |
| 105 |  | ANR32 | India |
| 106 | KJ603324 | BGR1 | India |
|  |  | BGR13 | India |
|  |  | BGR25 | India |
|  | KJ603346 | OTR3 | India |
|  |  | OTR8 | India |
| 107 | KJ603325 | BGR2 | India |
|  | KJ603327 | BGR4 | India |
|  | KJ603328 | BGR5 | India |
|  | KJ603329  KP159540 | BGR6 | India |
|  |  | BGR14 | India |
|  |  | BGR24 | India |
| 108 | KP159541 | BGR7 | India |
|  | KP159542 | BGR8 | India |
| 109 | KP159543 | BGR9 | India |
|  | KP159544 | BGR10 | India |
|  |  | BGR11 | India |
|  |  | BGR12 | India |
|  | KJ603336 | KTR1 | India |
| 110 |  | BGR15 | India |
|  |  | BGR22 | India |
|  |  | BGR23 | India |
|  | KJ603342 | KTR7 | India |
|  |  | KTR9 | India |
|  |  | KTR13 | India |
|  |  | KTR19 | India |
|  |  | OTR20 | India |
|  |  | OTR23 | India |
|  |  | OTR25 | India |
| 111 |  | BGR16 | India |
|  |  | BGR17 | India |
|  |  | BGR19 | India |
|  |  | BGR20 | India |
|  |  | BGR21 | India |
|  |  | BGR26 | India |
|  | KJ603353 | THR6 | India |
|  |  | THR20 | India |
| 112 | KJ603330 | KDR1 | India |
|  | KJ603331 | KDR2 | India |
| 113 | KJ603332 | KDR3 | India |
|  | KJ603333 | KDR4 | India |
| 114 | KJ603334 | KDR5 | India |
|  | KJ603335 | KDR6 | India |
|  | KP159547 | KDR9 | India |
|  |  | KDR12 | India |
|  |  | KDR14 | India |
|  |  | KDR17 | India |
| 115 | KP159545 | KDR7 | India |
|  |  | KDR19 | India |
| 116 |  | KDR10 | India |
|  |  | KDR13 | India |
|  |  | KDR15 | India |
|  |  | KDR18 | India |
| 117 |  | KDR16 | India |
| 118 | KJ603337 | KTR2 | India |
|  | KJ603339 | KTR4 | India |
|  | KJ603340 | KTR5 | India |
|  | KP159549 | KTR12 | India |
|  |  | KTR16 | India |
|  |  | KTR18 | India |
|  |  | KTR20 | India |
|  |  | KTR21 | India |
|  |  | KTR22 | India |
|  |  | KTR23 | India |
|  |  | KTR25 | India |
| 119 | KJ603338 | KTR3 | India |
| 120 | KJ603341 | KTR6 | India |
|  |  | KTR10 | India |
|  |  | KTR24 | India |
| 121 | KJ603343 | KTR8 | India |
|  | KP159550 | OTR5 | India |
|  | KP159551 | OTR6 | India |
|  |  | OTR9 | India |
|  |  | OTR21 | India |
|  |  | OTR26 | India |
|  |  | OTR28 | India |
|  | KJ603352 | THR5 | India |
|  | KP159553 | THR7 | India |
| 122 | KP159548 | KTR11 | India |
|  |  | KTR14 | India |
|  |  | KTR15 | India |
|  |  | KTR17 | India |
| 123 | KJ603345 | OTR2 | India |
|  | KP159552 | OTR7 | India |
| 124 |  | OTR17 | India |
| 125 | KJ603348 | THR1 | India |
| 126 | KJ603349 | THR2 | India |
|  | KJ603350 | THR3 | India |
|  |  | THR12 | India |
|  |  | THR16 | India |
|  |  | THR19 | India |
|  |  | THR23 | India |
| 127 | KP159554 | THR8 | India |
| 128 | KP159555 | THR9 | India |
|  | KP159556 | THR10 | India |
|  |  | THR18 | India |
| 129 |  | THR11 | India |
|  |  | THR13 | India |
|  |  | THR14 | India |
|  |  | THR15 | India |
|  |  | THR17 | India |
|  |  | THR22 | India |
| 130 | OM472146 | **Rathap20*** | New Zealand |
| 131 | OM472147 | **Rathap21*** | New Zealand |

Supplementary Table S5

Genetic parameters of 14 microsatellite loci analysed in a sample of 133 *Rattus rattus* from central New Zealand. Number of alleles (N_a_), mean allelic richness based on 7 diploid individuals (A_r_), population gene diversity (H_s_), observed heterozygosity (H_o_), F_IS_ and F_ST_ values from Fstat 2.9.4. H_s_, H_o_, F_IS_ and F_ST_ were estimated by Nei’s estimator (Nei, 1987)

| **Locus** | **N_a_** | **A_r_** | **H_s_** | **H_o_** | **F_IS_** | **F_ST_** |
| --- | --- | --- | --- | --- | --- | --- |
| Rr68 | 2 | 1.390 | 0.045 | 0.038 | 0.153 | 0.040 |
| D5Rat83 | 9 | 4.227 | 0.651 | 0.655 | -0.007 | 0.123 |
| Rr107 | 19 | 7.889 | 0.809 | 0.820 | -0.014 | 0.098 |
| Rr22 | 4 | 3.396 | 0.399 | 0.411 | -0.029 | 0.409 |
| D15Rat77 | 10 | 5.858 | 0.651 | 0.597 | 0.083 | 0.212 |
| D16Rat81 | 6 | 3.744 | 0.383 | 0.385 | -0.004 | 0.441 |
| D11Mgh5 | 15 | 5.986 | 0.723 | 0.768 | -0.062 | 0.116 |
| Rr67 | 5 | 3.817 | 0.498 | 0.536 | -0.075 | 0.193 |
| Rr21 | 11 | 5.338 | 0.558 | 0.584 | -0.046 | 0.284 |
| Rr14 | 7 | 4.833 | 0.633 | 0.619 | 0.022 | 0.108 |
| Rr54 | 9 | 5.357 | 0.572 | 0.576 | -0.007 | 0.257 |
| D7Rat13 | 11 | 5.886 | 0.668 | 0.638 | 0.045 | 0.197 |
| D18Rat96 | 12 | 6.323 | 0.754 | 0.864 | -0.146 | 0.099 |
| Rr17 | 9 | 5.954 | 0.652 | 0.705 | -0.081 | 0.218 |

Supplementary Figure 1

Haplotype network of available MtDNA DLoop data Rattus rattus sampled around the world.


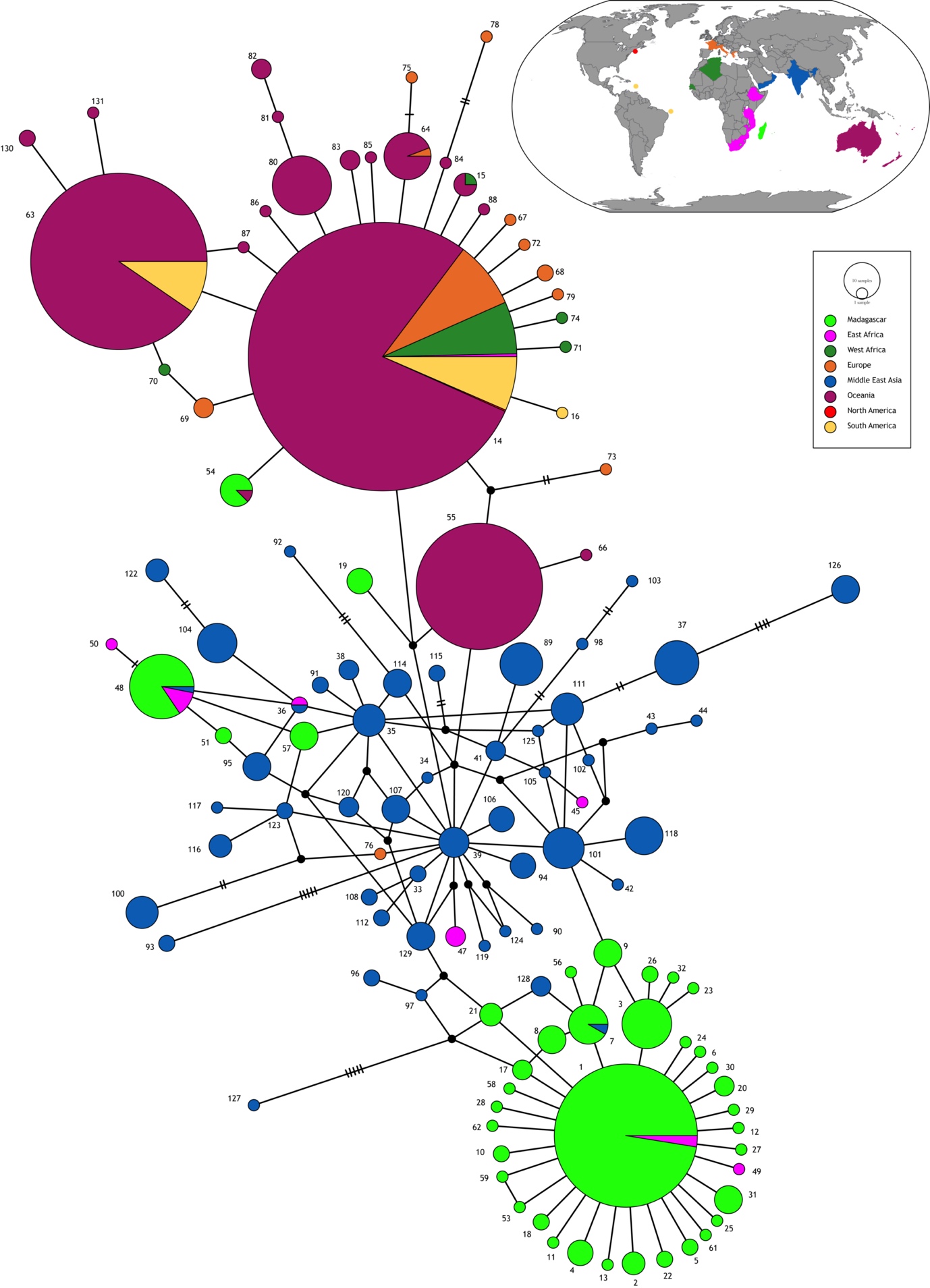


Supplementary Figure S2

Delta K for *Rattus rattus*: South Island (Picton) 14 loci, North Island (Wellington and Turitea) 14 loci, All 8 population 14 loci


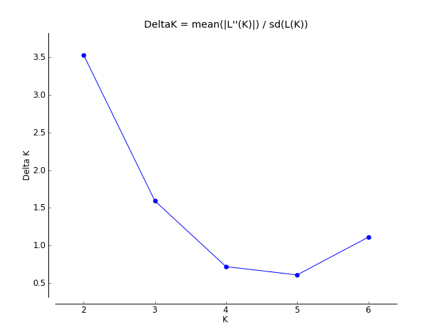

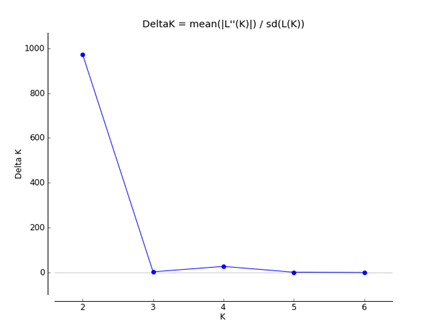

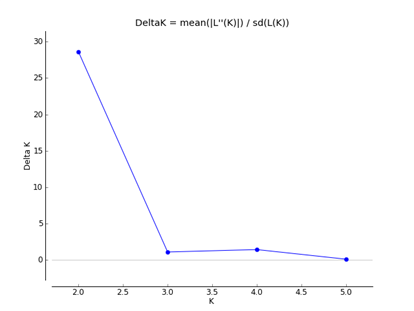


Supplementary Figure S3

Origin assignment for Ship rat, *Rattus rattus*. 14 microsatellite loci from 133 rats were used to perform GenePlot version 1.4: (A) Four North Island locations assigned to Belmont regional park and Turitea as the reference populations; (B) Pairwise assignment for Wedge Forest and Kaipupu Wildlife Sanctuary populations; (C) Pairwise assignment for Victoria Domain and Kaipupu Wildlife Sanctuary populations; (D) Pairwise assignment for Victoria Domain and Essons Valley populations. * indicates the individual who has missing microsatellite data.


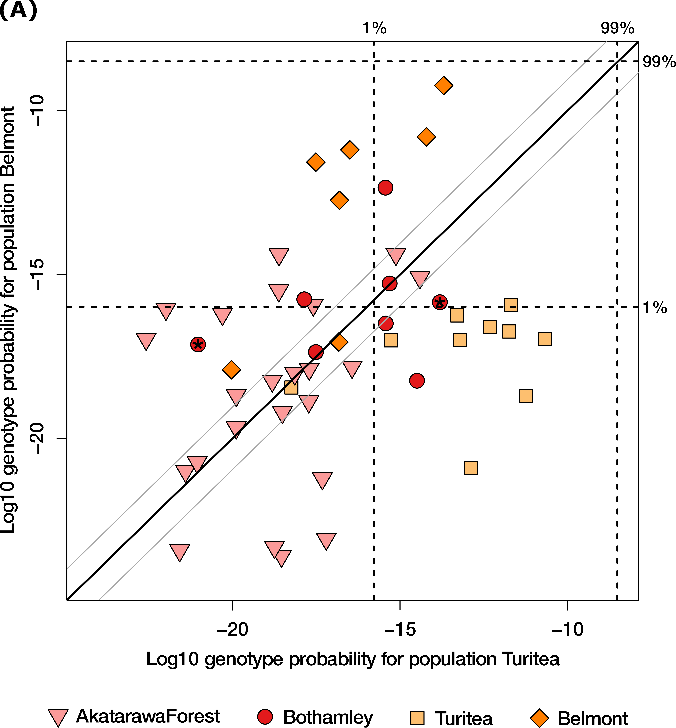

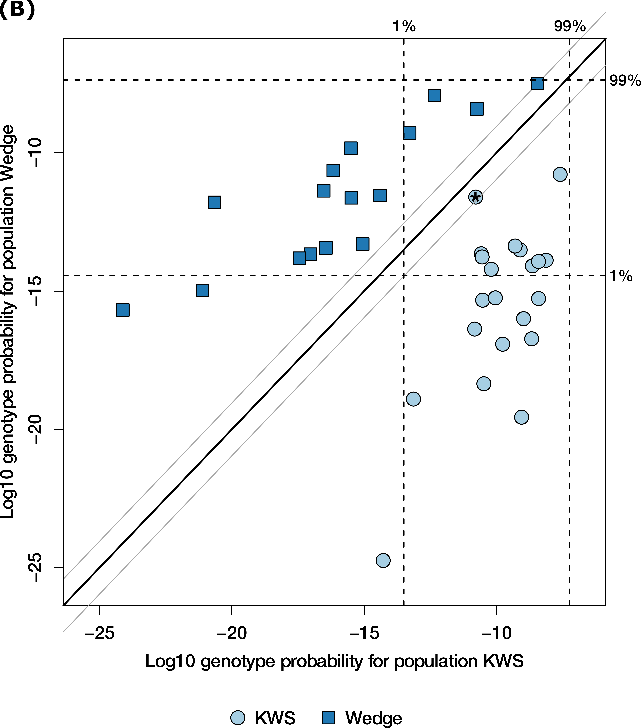


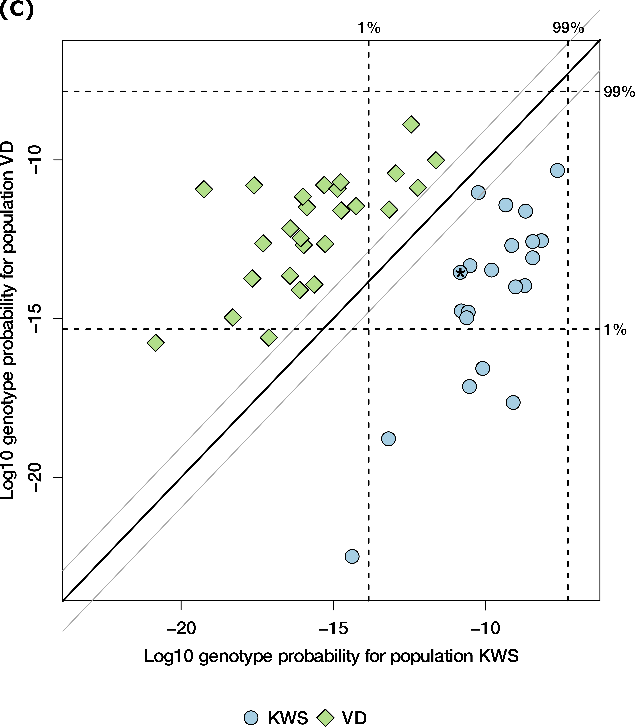

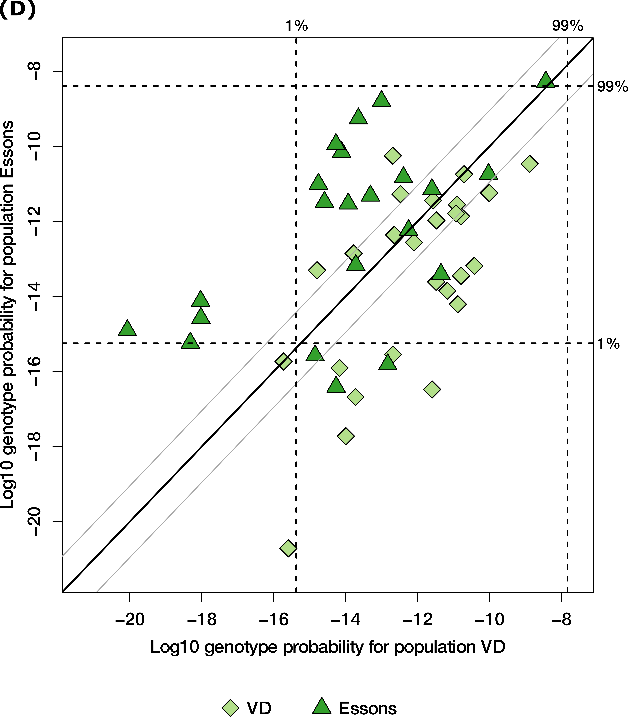


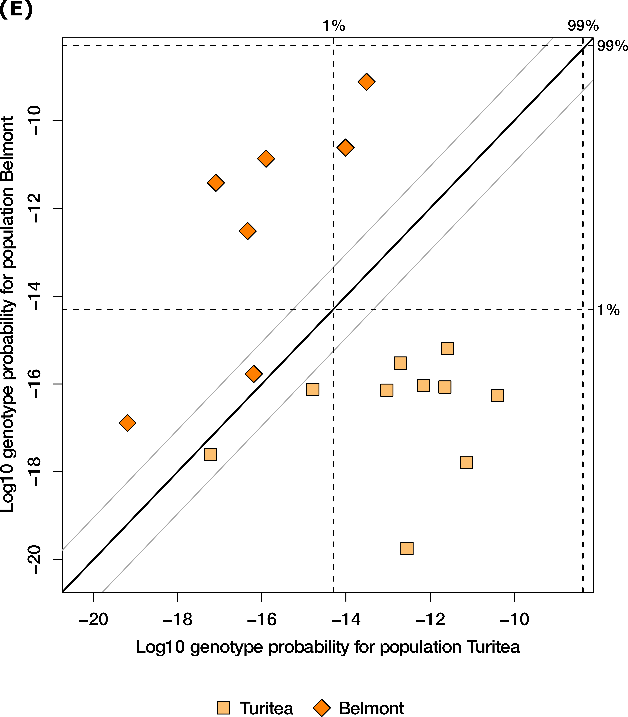

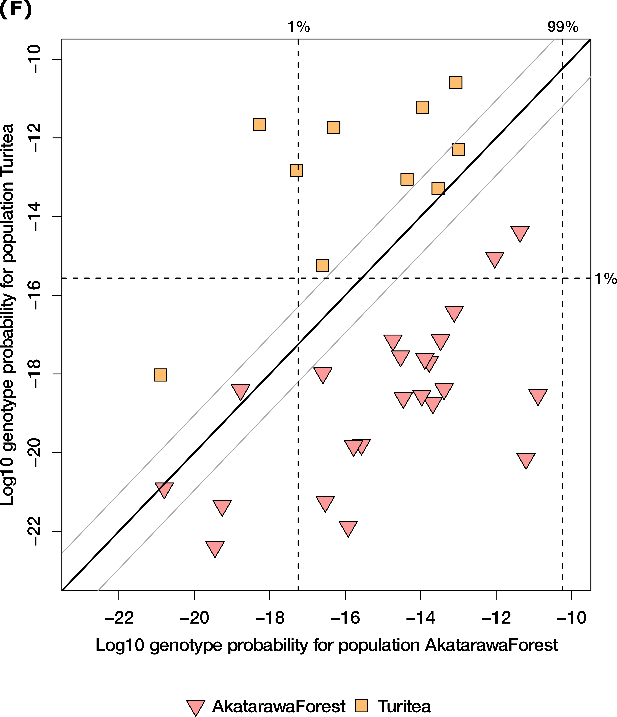


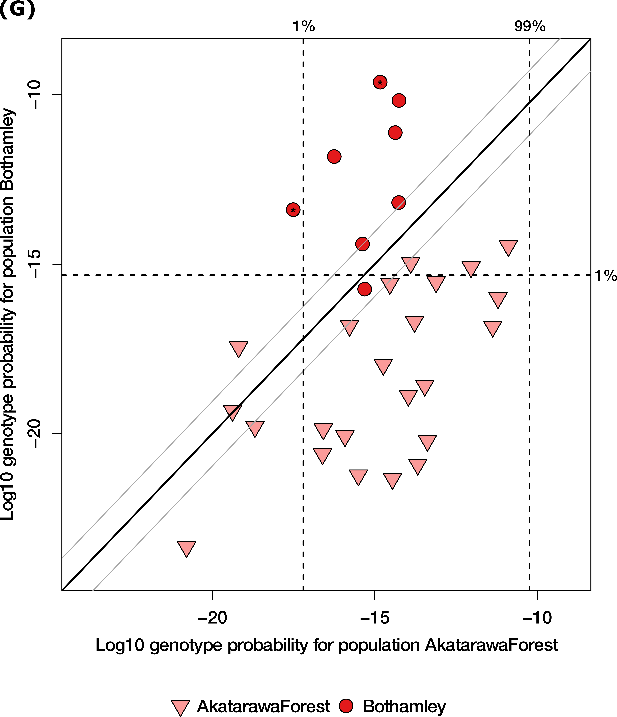

Supplement: Supplementary file 1 — Supplementary file1 (DOCX 800 KB) [file 10530_2023_3004_MOESM1_ESM.docx]
